# RAC1B function is essential for breast cancer stem cell maintenance and chemoresistance of breast tumor cells

**DOI:** 10.1038/s41388-022-02574-6

**Published:** 2023-01-05

**Authors:** Fuhui Chen, Sevim B. Gurler, David Novo, Cigdem Selli, Denis G. Alferez, Secil Eroglu, Kyriaki Pavlou, Jingwei Zhang, Andrew H. Sims, Neil E. Humphreys, Antony Adamson, Andrew Campbell, Owen J. Sansom, Cathy Tournier, Robert B. Clarke, Keith Brennan, Charles H. Streuli, Ahmet Ucar

**Affiliations:** 1 Manchester Breast Centre, Division of Cancer Sciences, School of Medical Sciences, Faculty of Biology, Medicine and Health, University of Manchester, Manchester, UK; 2 Wellcome Trust Centre for Cell Matrix Research, Faculty of Biology, Medicine and Health, University of Manchester, Manchester, UK; 3 Applied Bioinformatics of Cancer, Institute of Genetics and Cancer, University of Edinburgh Cancer Research Centre, Edinburgh, UK; 4 Breast Biology Group, Manchester Breast Centre, Division of Cancer Sciences, School of Medical Sciences, Faculty of Biology, Medicine and Health, University of Manchester, Manchester, UK; 5 Genome Editing Unit, Faculty of Biology, Medicine and Health, University of Manchester, Manchester, UK; 6 Cancer Research UK Beatson Institute, Glasgow, UK; 7 School of Cancer Sciences, University of Glasgow, Glasgow, UK

**Keywords:** Breast cancer, Cancer stem cells, Cancer models, Chemotherapy

## Abstract

Breast cancer stem cells (BCSC) are presumed to be responsible for treatment resistance, tumor recurrence and metastasis of breast tumors. However, development of BCSC-targeting therapies has been held back by their heterogeneity and the lack of BCSC-selective molecular targets. Here, we demonstrate that RAC1B, the only known alternatively spliced variant of the small GTPase RAC1, is expressed in a subset of BCSCs in vivo and its function is required for the maintenance of BCSCs and their chemoresistance to doxorubicin. In human breast cancer cell line MCF7, RAC1B is required for BCSC plasticity and chemoresistance to doxorubicin in vitro and for tumor-initiating abilities in vivo. Unlike Rac1, Rac1b function is dispensable for normal mammary gland development and mammary epithelial stem cell (MaSC) activity. In contrast, loss of Rac1b function in a mouse model of breast cancer hampers the BCSC activity and increases their chemosensitivity to doxorubicin treatment. Collectively, our data suggest that RAC1B is a clinically relevant molecular target for the development of BCSC-targeting therapies that may improve the effectiveness of doxorubicin-mediated chemotherapy.

## Introduction

Breast cancer is the most common cancer in women and the fourth leading cause of cancer-related deaths worldwide [1]. Despite the advances in treatment options for patients with breast cancer, tumor recurrence and therapy resistance are still significant and contribute to high mortality rates. Breast cancer stem cells (BCSC), also known as tumor-initiating cells, are presumed to be responsible for therapy resistance, tumor recurrence and metastasis. Therefore, BCSC-targeted therapies could have the potential to improve clinical outcomes. However, the development of such therapies is complicated by the BCSC heterogeneity, driven by the intrinsic stem cell plasticity, and the lack of knowledge on BCSC-specific molecular targets that are dispensable for normal adult stem cells [2].

RAC1 is a small GTPase that functions as a key signaling node downstream of various microenvironmental signaling pathways, including those triggered by cell adhesion and growth factors. RAC1 signaling is upregulated in various cancers, including breast cancer [3–7], and regulates cellular processes such as tumor cell survival, proliferation and invasion [8]. Importantly, RAC1 is implicated in therapy resistance of tumor cells against both cytoablative [9–11] and targeted treatments [9, 12–16]. However, due to its almost ubiquitous expression and critical functions in various organ systems [17–20], there is little clinical relevance of potential RAC1-targeted treatments. Hyperactivation of RAC1 signaling in tumors is associated with rare mutations, upregulated expression or misregulation by RAC1-regulatory proteins including Guanine-nucleotide exchange factors (GEFs), GTPase-activating proteins (GAPs) and Guanine-nucleotide dissociation inhibitors (GDIs) [7, 8, 21–23].

Hyperactivation of RAC1 signaling in some solid tumors is in part due to the alternative splicing of *RAC1* to generate the RAC1B variant, a constitutively active form of the small GTPase [24–26]. RAC1B has an additional exon (i.e., exon3b) encoding 19 amino acids with an in-frame insertion just after its Switch-II domain. This leads to a structural change favoring the active GTP-bound state independent of GEF-mediated activation [27].

Here, using human breast cancer cell line MCF7, we showed that RAC1B function is essential in BCSCs for their plasticity, chemoresistance to doxorubicin treatment and tumor-initiating abilities. Using genetically engineered mouse models, we determined that in HER2/Neu-driven mammary tumors Rac1b is expressed by a substantial subset of BCSCs, which require Rac1b function for their maintenance/activity, and the loss-of Rac1b function sensitizes them to the chemotherapeutic effect of doxorubicin treatment. Furthermore, we demonstrated that, unlike Rac1, Rac1b function is dispensable for mammary epithelial stem cells (MaSCs) and normal mammary gland development or function, thus suggesting clinical feasibility of RAC1B-targeting. Finally, TCGA dataset analysis revealed that higher RAC1B expression levels in breast tumors predict worse overall survival in doxorubicin-treated patient groups, thus providing clinical confirmation to our findings in the experimental models of breast cancer. Taken together, our results propose RAC1B as a promising BCSC-specific molecular target to sensitize the RAC1B-expressing chemoresistant breast tumors to the therapeutic effects of doxorubicin treatment.

## Results

### BCSCs require RAC signaling for their self-renewal maintenance

**Fig. 1:**
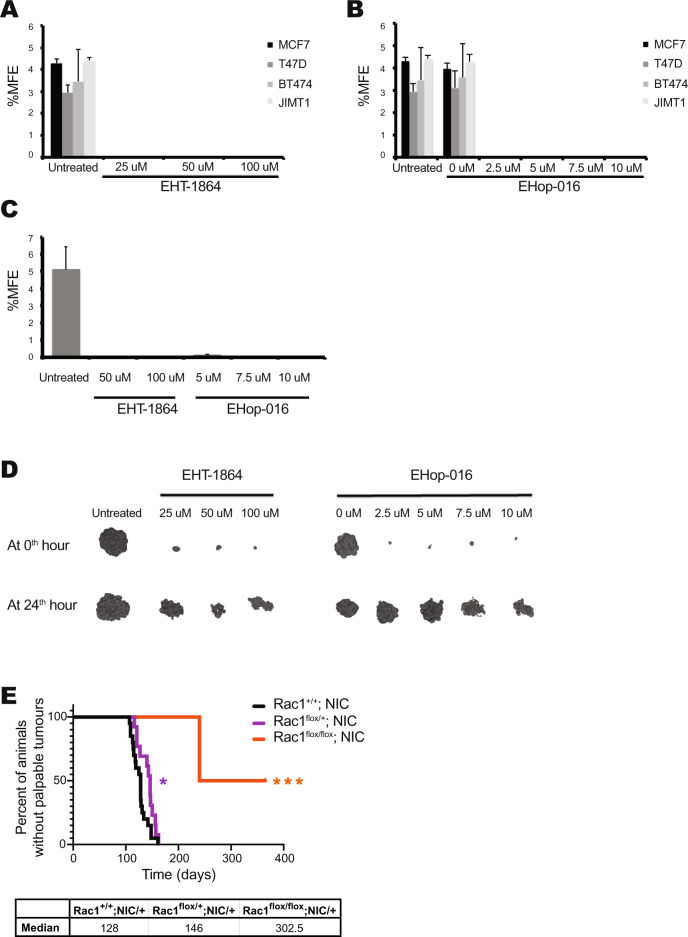
RAC activity is required for BCSCs in vitro and breast tumorigenesis in vivo. **A**, **B** Mammosphere-forming efficiency (%MFE) of human breast cancer cell lines (MCF7, T47D, BT474, and JIMT1) in the presence of RAC-specific inhibitors EHT-1864 (**A**) and EHop-016 (**B**) at the indicated concentrations in culture. Values represent the mean ± SD of 3 independent experiments. **C** Mammosphere-forming efficiency (%MFE) for the secondary mammosphere culture of MCF7 cells, which were treated with the indicated concentrations of EHT-1864 or EHop-016 only during their primary mammosphere culture. Values represent the mean ± SD of 3 independent experiments. **D** Representative images of structures formed by MCF7 cells that were treated with the indicated concentrations of EHT-1864 or EHop-016 in the primary mammosphere culture either starting from the time of plating (0 h) or 24^th^ hour after plating. In (**A**), (**B**), and (**D**), the ‘0 uM’ EHop-016 treatment group corresponds to vehicle-only control, which is the same DMSO concentration as in ’10 uM’ treatment group. **E** Kaplan–Meier graphs for the tumor latency in Rac1^+/+^;MMTV-NIC (*n* = 20), Rac1^flox/+^;MMTV-NIC (*n* = 13), Rac1^flox/flox^;MMTV-NIC (*n* = 2) mice. Time represents postnatal age in days. Median age of palpable tumor formation for each genotype is shown below the graph (*p < 0.05, ***p < 0.01; Lox-rank Mantel Cox test).

We have previously shown that Rac1 is required for MaSC self-renewal [18]. To elucidate whether RAC signaling is also required for BCSC activity, we used two specific RAC-inhibitors with different modes of action in the mammosphere culture of human breast cancer cell lines that are known to generate proliferation-driven mammospheres [28] and represent different breast cancer subtypes: Luminal-A (MCF7 and T47D), Luminal-B (BT474), and HER2 + (JIMT-1). The inhibitors render RAC proteins in a nucleotide-free inactive state (EHT-1864) or prevent their activation by GEFs (EHop-016) [29, 30]. Interestingly, the mammosphere-forming ability of these cell lines was completely abrogated in the presence of either EHT-1864 or EHop-016 (Fig. 1A, B).

Mammosphere formation requires an initial self-renewing division of the stem cell, followed by consecutive rounds of proliferation of their non-stem-cell progeny [31]. Therefore, we asked whether RAC inhibition results in BCSC depletion or inhibition of cell proliferation, both of which could prevent mammosphere formation. To address this question, we treated MCF7 cells with RAC inhibitors in the primary mammosphere culture for 5 days and then performed a secondary mammosphere formation assay in the absence of inhibitors. If the effect of RAC inhibition on primary mammosphere formation is due to inhibition of cell proliferation, BCSCs would be expected to form mammospheres during secondary mammosphere culture when RAC inhibitors are removed. Our results demonstrated that MCF7 cells with inhibited RAC signaling during their primary mammosphere culture did not form any secondary mammospheres despite the absence of RAC inhibitors (Fig. 1C), suggesting that their BCSC pool was depleted during the primary mammosphere culture. Given that these RAC-inhibitors do not cause any significant cytotoxicity of MCF7 cells at the concentrations we used (Supplementary Fig. 1), our results indicate that inhibition of RAC signaling specifically leads to BCSC depletion rather than inducing a wider cellular cytotoxicity.

We then tested whether RAC inhibition also affects the proliferation of non-stem cell progenies of BCSCs by initiating RAC inhibition in the mammosphere culture of MCF7 cells either at 24 h after plating or at the time of plating. Our results showed that the effect of RAC inhibition is restricted to the initial cell divisions of BCSCs that take place within the first 24 h of culture, whereas the proliferation of non-stem cell progeny of BCSCs does not rely on RAC signaling (Fig. 1D). However, high concentrations of RAC inhibitors led to a cell-shedding phenotype, which was also observed when fully formed mammospheres at Day-5 of culture were treated with high concentrations of these inhibitors (data not shown), suggesting a potential inhibition of cell-cell adhesion in the presence of high inhibitor concentrations.

Since BCSCs are essential for breast tumorigenesis, we examined whether Rac1 is required for breast tumorigenesis in vivo. We generated a double transgenic mouse line bearing floxed-*Rac1* allele [32] and MMTV-Neu-IRES-Cre (NIC) transgene [33], which allows genetic deletion of *Rac1* in Neu-overexpressing cells. Latency analysis of palpable tumor formation revealed a significant delay in heterozygous Rac1^flox/+^;MMTV-NIC mice compared with Rac1^+/+^;MMTV-NIC mice (Fig. 1E). Although we were able to obtain only two Rac1^flox/flox^;MMTV-NIC females, only one of them developed palpable tumors during its first year of age (Fig. 1E). Since Rac1 is indispensable for early-stage embryogenesis [34], we suspect that leaky expression from the MMTV promoter during early embryogenesis may have led to *Rac1* deletion and thus embryonic lethality in most Rac1^flox/flox^;MMTV-NIC embryos.

Taken together, our results reveal that RAC signaling is required for the self-renewal maintenance of BCSCs in vitro, and that loss-of Rac1 function delays or suppresses breast tumorigenesis in a dose-dependent manner in vivo.

### RAC1B is involved in the regulation of BCSC plasticity in MCF7 cells

RAC inhibition or genomic deletion of *Rac1* results in the loss of both RAC1 and RAC1B functions [35, 36]. Therefore, we decided to investigate to which extent the observed phenotypes would be recapitulated by targeting RAC1B alone. ER + cell lines MCF7, T47D and BT474 were found to express RAC1B, albeit at different levels (Supplementary Fig. 2). In mice, Rac1b mRNA was detected predominantly in basal mammary epithelial cells of mice at nulliparous or early-pregnancy stages as well as in the tumor cells of the MMTV-NIC mouse model (Supplementary Fig. 2).

**Fig. 2:**
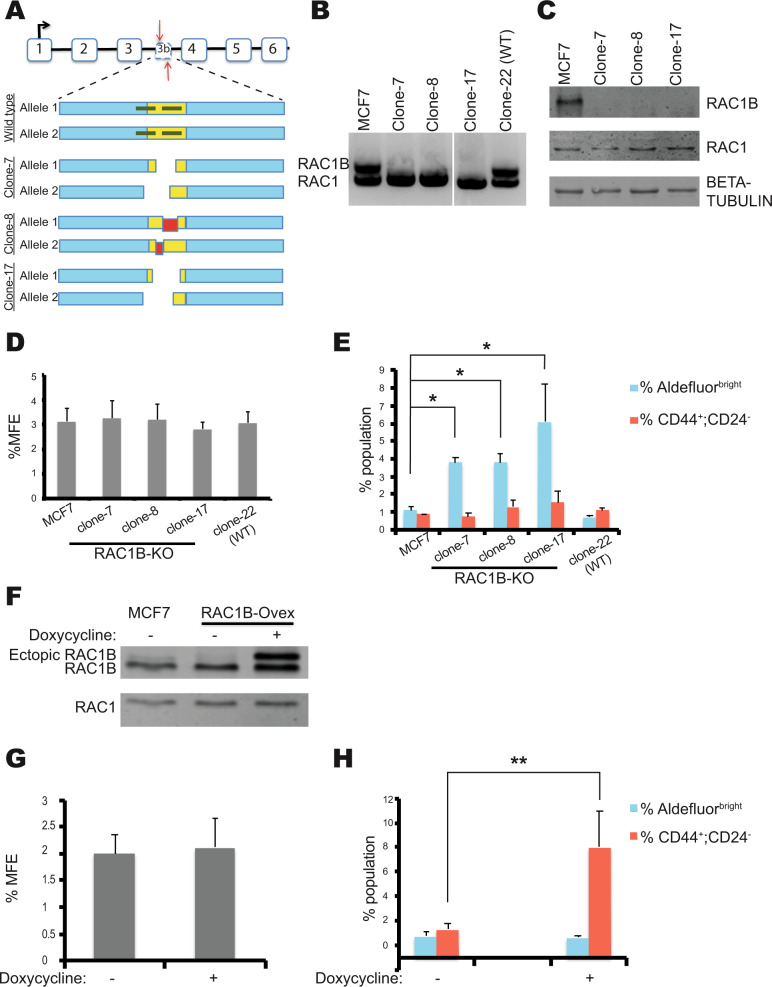
RAC1B regulates BCSC plasticity in MCF7 cells. **A** Schematic presentation of CRISPR/Double nickase targeting strategy and allelic maps showing indel mutations generated in each independent MCF7 single-cell clone. Vertical red arrows on the upper image of exon map of *RAC1* gene and the green horizontal lines in the allele map of wildtype clone marks the targeted genomic sites by sgRNA sequences used. In allelic maps, the exon3b sequence is depicted in yellow and the flanking intronic sequences in blue; gaps correspond to deletions, whereas the regions shown in red corresponds to insertions. **B**, **C** RT-PCR (**B**) and immunoblot analysis (**C**) of single-cell clones. Beta-tubulin was used as a loading control in immunoblot experiments. **D** Mammosphere-forming efficiency (%MFE) of parental MCF7 and single-cell clones. Values represent the mean ± SD of 3 independent experiments. No significant difference between clones was observed as determined by two-tailed paired *t*-test. **E** Percentage of cells that form Aldefluor^bright^ or CD44^+^;CD24^-^ subpopulations in parental MCF7 and single-cell clones as determined by flow cytometry analyses. Values represent the mean ± SD of 3 biological replicates. *p < 0.05; one-tailed paired *t*-test for comparison of each clone to parental MCF7 sample. **F** Immunoblot analysis of RAC1 and RAC1B expression for the stable-transgenic MCF7 clone with doxycycline-inducible RAC1B overexpression that are treated with or without 2 ug/ml doxycycline. **G** Mammosphere-forming efficiency (%MFE) of the stable-transgenic MCF7 clone with doxycycline-inducible RAC1B overexpression that are treated with or without 2 ug/ml doxycycline. Values represent the mean ± SD of 3 independent experiments. No significant difference was observed as determined by two-tailed paired *t*-test. **H** Percentage of cells that form the Aldefluor^bright^ and CD44^+^;CD24^-^ subpopulations in the stable-transgenic MCF7 clone with doxycycline-inducible RAC1B overexpression that are treated with or without 2 ug/ml doxycycline (**p < 0.01; one-tailed paired *t*-test).

To determine whether variant-specific loss-of RAC1B affects BCSCs, we employed CRISPR/Double-nickase method to target the exon3b-coding genomic sequence in MCF7 cells followed by single-cell cloning to ensure genomic homogeneity for further phenotypic analyses. Several single-cell clones were obtained that specifically lacked RAC1B mRNA and protein (Fig. 2B, C) and sequencing of their genomic DNA revealed distinct insertion/deletion (indel) mutations in each allele of each clone (Fig. 2A). Interestingly, even small deletions within the exon3b-coding sequence resulted in the loss of *RAC1B* mRNA, suggesting a disruption of splicing-regulatory sequences required for *RAC1B* splicing.

We found that the loss-of RAC1B function in these MCF7 clones did not alter their primary or secondary mammosphere-forming capacity (Fig. 2D and Supplementary Fig. 3A), although it caused a significant increase in the frequency of their Aldefluor^bright^ BCSC population as determined by flow cytometry (Fig. 2E). To address whether gain-of RAC1B function leads to an inverse phenotype, we generated stable-transgenic MCF7 cells with doxycycline-inducible expression of RFP-RAC1B fusion protein (Fig. 2F). Like RAC1B-null MCF7 clones, the RAC1B overexpression did not alter primary or secondary mammosphere-forming capacity of MCF7 cells (Fig. 2G and Supplementary Fig. 3B). However, RAC1B overexpression led to a significant increase in their CD44^+^;CD24^-^ BCSC population (Fig. 2H).

Earlier studies have described Aldefluor^bright^ and CD44^+^;CD24^-^ populations in MCF7 cells as the proliferative epithelial-like and quiescent mesenchymal-like states of BCSCs, respectively, and suggested that the ability to reversibly transit between these states underlies the plasticity within the BCSC pool [37, 38]. Our results therefore suggest that RAC1B regulates the reversible switching between epithelial-like and mesenchymal-like states of BCSCs without altering total BCSC numbers, and it is likely to be required for the mesenchymal-like BCSC state.

### RAC1B function is essential for the chemoresistance of MCF7 cells via regulating BCSC self-renewal/maintenance and plasticity

**Fig. 3:**
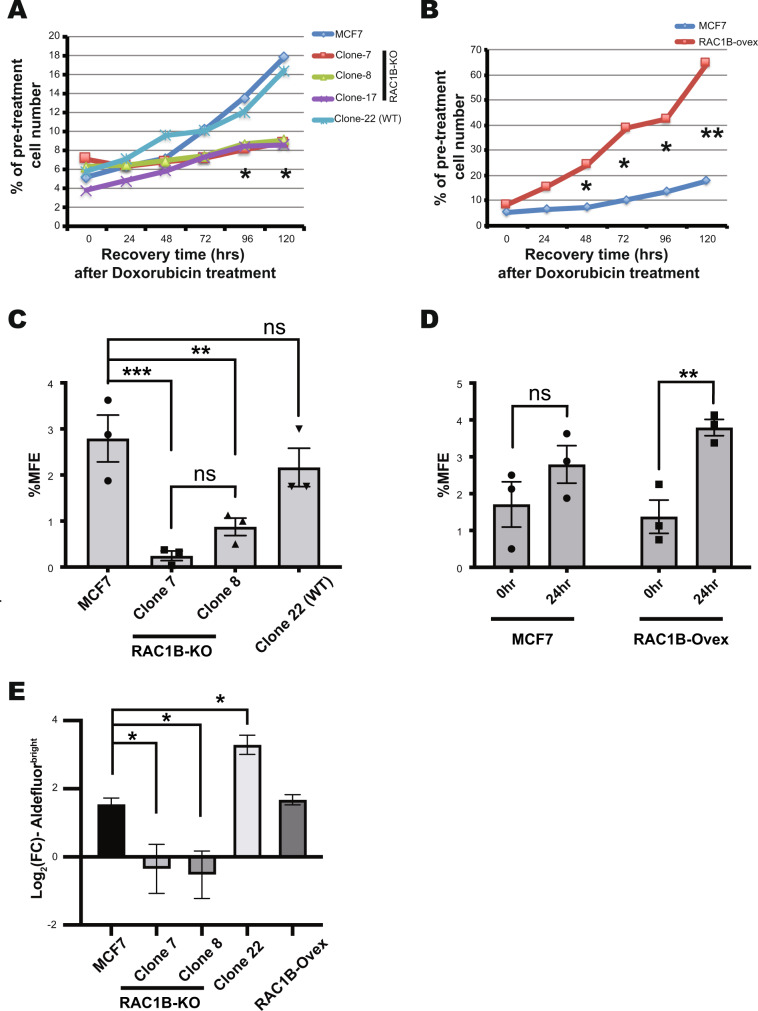
RAC1B function is essential for the BCSC maintenance in response to doxorubicin treatment in MCF7 cells. **A** Cell growth curve of parental MCF7, RAC1B-proficient Clone 22 (WT) and RAC1B-null single-cell clones in post-treatment recovery period after 2.5 uM doxorubicin treatment for 24 h. Cell numbers are presented as percentage of the pre-treatment cell number for each individual clone. Each data point represents the mean of 3 independent experiments. Significant differences were observed for all 3 RAC1b-null clones compared to parental MCF7 at 96- and 120-h post-treatment recovery time (*p < 0.05; two-tailed unpaired *t*-test). **B** Cell growth curve of stable-transgenic MCF7 clone with doxycycline-inducible RAC1B overexpression continuously treated with (RAC1B-ovex) or without (MCF7) 2.5 uM doxycycline in post-recovery period after 2.5 uM doxorubicin treatment for 24 h. Percentage cell number calculations are as described in (**A**). Each data point represents the mean of 3 independent experiments. Significant differences were observed at 48-, 72-, 96- and 120-h post-treatment recovery time (*p < 0.05; **p < 0.01; two-tailed unpaired *t*-test). **C** Mammosphere-forming efficiency (%MFE) of parental MCF7 and single-cell clones when plated in mammosphere culture 24 h after the end of 2.5 uM doxorubicin treatment. Bar graphs represent the mean ± SEM of 3 independent experiments with individual values shown. (**p < 0.01; ***p < 0.005; one-way ANOVA). **D** Mammosphere-forming efficiency (%MFE) of parental MCF7 and RAC1B-ovex MCF7 clone when plated in mammosphere culture 0 or 24 h after the end of 2.5 uM doxorubicin treatment. Bar graphs represent the mean ± SEM of 3 independent experiments with individual values shown. (**p < 0.01; one-way ANOVA). **E** Fold change in the size of Aldefluor^bright^ cell subpopulation in parental MCF7, RAC1B-proficient, RAC1B-null or RAC1B-overexpressing clones 24 h after the end of 2.5 uM doxorubicin treatment compared with their own untreated control groups. Fold change differences are shown in Log_2_ scale and represent the mean ± SEM of 3 independent experiments. (*p < 0.05; one-way ANOVA).

Resistance to chemotherapy is a feature often attributed to CSCs. As cytoablative treatments specifically target proliferating cells, the chemoresistant population of BCSCs is likely to be the CD44^+^;CD24^-^ BCSCs, which were identified to be more quiescent than Aldefluor^bright^ cells in earlier studies [37, 38]. Given that CD44^+^;CD24^-^ BCSC subpopulation may require RAC1B function, we hypothesized that RAC1B may have crucial roles in chemoresistance. We therefore determined the effect of doxorubicin, a chemotherapeutic agent commonly used for the treatment of patients with breast cancer, on RAC1B-null and RAC1B-overexpressing MCF7 cells. We treated these cells with 2.5 uM doxorubicin for 24 h, which led to more than 90% of cell loss, and then measured the recovery as cell growth in the absence of doxorubicin. Parental MCF7 and RAC1B-proficient MCF7 clone (Clone-22) showed a slow but steady recovery during the five-day period after doxorubicin removal (Fig. 3A). In contrast, RAC1B-null MCF7 clones did not recover during the same period (Fig. 3A) nor up to 3 weeks post-treatment (data not shown). Conversely, the RAC1B-overexpressing cells showed a robust recovery upon doxorubicin withdrawal (Fig. 3B) compared with the same cells not treated with doxycycline to induce RAC1B overexpression. These results indicate that RAC1B function is required for the chemoresistance of MCF7 cells in vitro.

Next, we have analyzed the BCSC populations in these doxorubicin-treated cells to determine whether the RAC1B function in regulating BCSC plasticity might explain the observed phenotypes. First, we determined the mammosphere-forming efficiency in these doxorubicin-treated cells at 0 or 24 h of post-treatment recovery period. Our results demonstrated that RAC1B-null MCF7 clones had a significantly reduced BCSC frequency compared to parental MCF7 cells at 24 h after doxorubicin treatment (Fig. 3C). Although RAC1B-overexpressing MCF7 cells had similar BCSC frequencies as the parental MCF7 cells post-treatment (i.e., 0 h), they had a significantly higher increase in their BCSC frequency during the initial 24 h recovery period (Fig. 3D). These results suggest that RAC1B function is essential for the survival and/or re-population of BCSCs after doxorubicin treatment.

To further evaluate the effects of doxorubicin treatment on the BCSC plasticity, we have analyzed the changes within the Aldefluor^bright^ and CD44^+^;CD24^-^ populations in these cells. Parental MCF7 cells, the wildtype MCF7 clone and the RAC1b-overexpressing MCF7 cells had an increased frequency of Aldefluor^bright^ cell population at 24 h post-treatment compared to their non-treated control groups (Fig. 3E). In contrast, the Aldefluor^bright^ cell population frequency was decreased in RAC1B-null MCF7 clones compared to their own non-treated control groups (Fig. 3E), suggesting that the post-treatment re-population of the proliferative epithelial-like BCSC population was probably impaired in the absence of RAC1B function. This may explain why RAC1B-null MCF7 clones failed to recover their cell growth after doxorubicin treatment (Fig. 3A). Interestingly, the CD44^+^;CD24^-^ populations in the parental MCF7 and wildtype MCF7 clone displayed a reduction after 24 h doxorubicin treatment followed by a sharp increase during the post-treatment recovery period (Supplementary Fig. 4). In contrast, the RAC1B-null and RAC1B-overexpressing MCF7 clones had either a slight increase or no change in their CD44^+^;CD24^-^ populations during doxorubicin treatment, which was again followed by a sharp increase during the post-treatment recovery (Supplementary Fig. 4). The increased size of CD44^+^;CD24^-^ populations 24 h post-treatment in all cell lines irrespective of their RAC1B expression status is likely due to the doxorubicin-induced epithelial-mesenchymal transition (EMT) as reported for MCF7 cells earlier [39, 40].

Taken together, these results suggest that MCF7 cells require RAC1B function for their chemoresistance to doxorubicin treatment and the role of RAC1B function in regulating BCSC plasticity may ensure the BCSC maintenance and/or self-renewal during and after doxorubicin treatment, respectively.

### RAC1B function is essential for in vivo tumor initiating ability of MCF7 cells

**Fig. 4:**
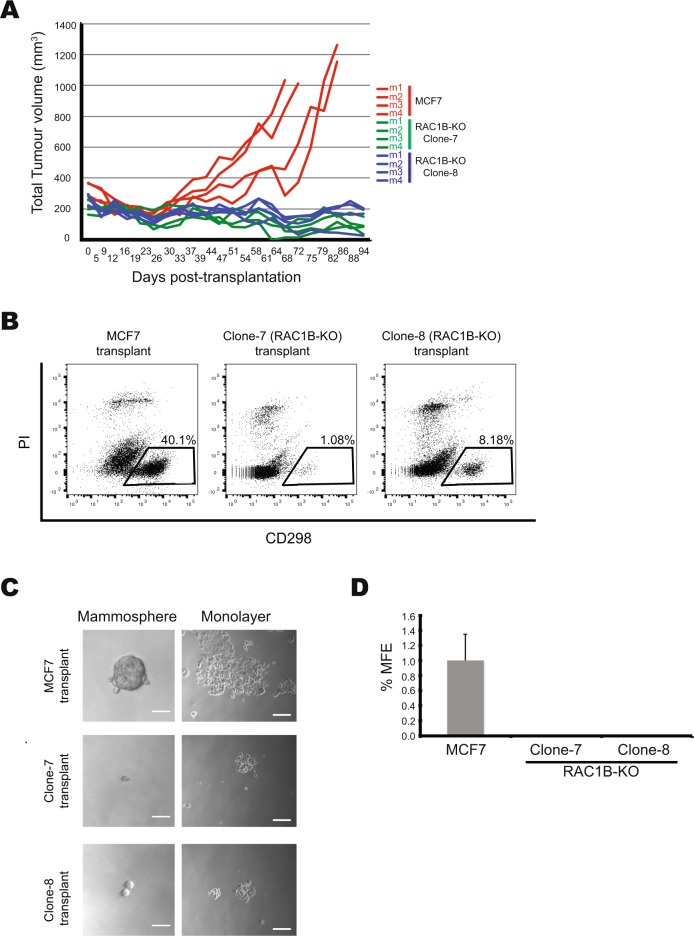
RAC1B function is required for the tumor-initiating abilities of BCSC in MCF7 cells. **A** In vivo tumor growth capacity of parental MCF7 and two RAC1B-null single-cell clones were determined by xenograft transplantation assay by implanting 100,000 cells into both right and left dorsal sides of 4 immunodeficient nude mice per group, and tumor volumes were measured twice weekly with calipers. The data represent the total tumor volume for each individual mouse. **B** Flow cytometry analysis of the explants isolated from mice shown in (**A**). Representative dot plots show single cells stained with propidium iodide (PI) as a dead cell marker and CD298 as a human-specific antigen used for distinguishing the cells of human origin. **C** Representative images of mammosphere and monolayer cultures of CD298^+^ cells sorted from explants as shown in (**B**). Scale bars represent 50 um and 100 um for mammosphere and monolayer cultures, respectively. **D** Mammosphere-forming efficiency (%MFE) of CD298^+^ cells sorted from explants as shown in (**B**). Of note, the data for MCF7 explants is the mean ± SD for 3 animals, whereas for Rac1B-null clones the data represents %MFE of pooled cell samples from explants of the same single-cell clone.

Although the importance of BCSC plasticity in tumor-initiating ability of BCSCs is largely unknown, it is likely to play crucial roles in long-term maintenance of BCSCs while generating large numbers of new tumor cells. Therefore, we investigated whether RAC1B is required for tumor-initiating ability of BCSCs in vivo. Xenograft transplantation of parental MCF7 cells resulted in tumor formation within 6–7 weeks, whereas RAC1B-null MCF7 clones formed no visible tumors even up to 100 days post-transplantation (Fig. 4A). At the experimental endpoint (either maximum tumor burden of 1.25 cm^3^ or 100 days post-transplantation), tumors/tissues at the site of transplantation were dissected and analyzed by flow cytometry for the human-specific antigen CD298 expression (Fig. 4B). Surprisingly, explants obtained from mice transplanted with RAC1B-null MCF7 clones still contained some CD298^+^ cells, despite the absence of tumor growth. However, unlike parental MCF7 cells recovered from xenograft tumors, RAC1B-null MCF7 cells sorted as CD298^+^ population from those explants neither formed mammospheres nor monolayer colonies (Fig. 4C, D). These results demonstrate that RAC1B is indispensable for BCSC self-renewal and tumor growth in vivo.

### Loss of Rac1b function does not alter mammary gland development

Rac1 is indispensable for mammary gland development and function, particularly in MaSCs in nulliparous animals, lobuloalveolar development during pregnancy and tissue remodeling during involution [18, 41–44]. Since the Rac1^flox/flox^ mouse line used in these studies result in the loss of both Rac1 and Rac1b, we generated a Rac1b^−/−^ mouse line to study Rac1b-specific loss-of-function phenotypes (Supplementary Fig. 5). In both C57BL/6 and FVB backgrounds, Rac1b^−/−^ mice were born with expected Mendelian ratios and had a normal life span with no apparent health problems. Immunoblot analysis of mammary gland tissues of Rac1b^−/−^ mice at lactation and involution stages has shown that genomic deletion of exon3b-encoding region has not impaired Rac1 expression (Supplementary Fig. 5C, D).

**Fig. 5:**
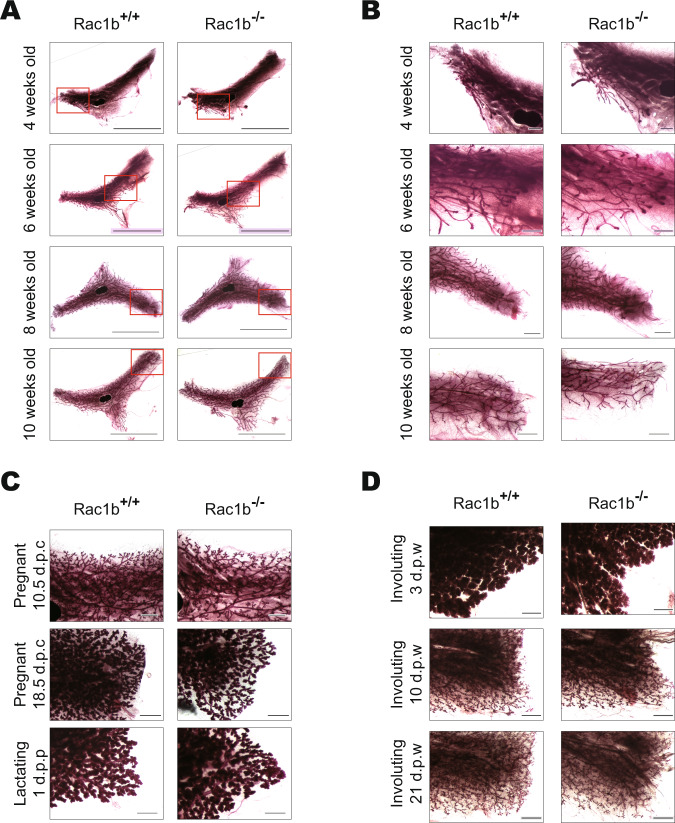
Rac1b is dispensable during mammary gland development. **A** Representative images of whole-mount stained No:4 inguinal mammary glands of 4-, 6-, 8-, and 10-week-old nulliparous Rac1b^+/+^ and Rac1b^−/−^ mice. **B** Higher magnification images of distal parts of the ductal tree at locations depicted with red rectangles in (**A**). **C** Representative images of whole-mount stained No:4 inguinal mammary glands of Rac1b^+/+^ and Rac1b^−/−^ mice at developmental stages of early pregnancy (10.5 d.p.c [days post-coitum]), late pregnancy (18 d.p.c.), and lactation (Day 1 d.p.p [days post-partum]). **D** Representative images of whole-mount stained No:4 inguinal mammary glands of Rac1b^+/+^ and Rac1b^−/−^ mice at involution stages of 3, 10 and 21 days post-weaning (d.p.w). Scale bars represent 1 cm in (**A**, **C**, **D**) and 1 mm in (**B**). Images shown are representative of *n* ≥ 3 mice.

To determine whether the loss-of Rac1b function hampers mammary gland development, we performed whole-mount staining of mammary glands obtained from mice at different postnatal developmental stages. During pubertal stages, there were no macroscopically obvious differences in ductal outgrowth or branching between Rac1b^−/−^ and Rac1b^+/+^ glands (Fig. 5A, B). Similarly, Rac1b^−/−^ glands were indistinguishable from Rac1b^+/+^ glands in early and late pregnancy, lactation, and involution stages (Fig. 5C, D).

Next, we evaluated whether Rac1b deficiency would affect mammary epithelial lineage diversification and/or MaSC activities. Basal (CD49f^high^;CD24^low^) and luminal (CD49f^low^;CD24^high^) epithelial cell populations showed a similar distribution within the glands of 8-week-old nulliparous Rac1b^−/−^, Rac1b^+/−^ and Rac1b^+/+^ mice as determined by flow cytometry (Supplementary Fig. 6A–C). When sorted and plated in mammosphere culture, Rac1b^−/−^ luminal and basal epithelial populations were indistinguishable from their Rac1b^+/+^ counterparts in terms of luminal progenitor-driven acini and MaSC-driven mammosphere formation, respectively (Supplementary Fig. 6D, E). These results indicate that Rac1b function is dispensable for both luminal progenitor and MaSC activities.

Together, our data demonstrate that mammary gland phenotypes of Rac1-null mice [18, 41, 42] are due to the loss-of-function of Rac1, but not Rac1b. Importantly, Rac1b deficiency results in no obvious alterations in MaSCs or defects in normal mammary gland development/function.

### Rac1b expression marks a substantial subset of BCSCs and is required for BCSC maintenance

**Fig. 6:**
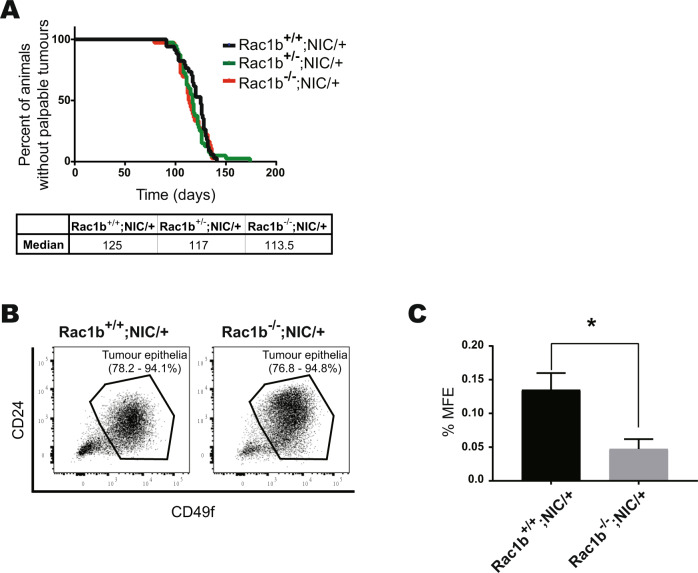
Loss-of Rac1b function results in decreased BCSC frequency without altering tumor latency. **A** Kaplan–Meier graphs of tumor latency in Rac1b^+/+^;MMTV-NIC (*n* = 34), Rac1b^+/−^;MMTV-NIC (*n* = 40), Rac1b^−/−^;MMTV-NIC (*n* = 36) mice. Time represents postnatal age in days. Median age of palpable tumor formation for each genotype is shown below the graph. There were no statistically significant differences between genotypes according to the Lox-rank Mantel Cox test. **B** Representative dot plots of CD49f versus CD24 expression for flow cytometry analysis of primary mammary tumor cells obtained from Rac1b^+/+^;MMTV-NIC and Rac1b^−/−^;MMTV-NIC mice are shown for the single, alive, lineage-negative cell population. The observed range for tumor epithelial cell frequency in tumors of different animals of same genotype is shown above the respective gate (*n* = 9). **C** Mammosphere forming efficiency (%MFE) of tumor epithelial cell populations sorted from Rac1b^+/+^;MMTV-NIC and Rac1b^−/−^;MMTV-NIC tumors as shown in (**B**). Values represent the mean ± SEM of 5 animals. (*p < 0.05; paired *t*-test).

Dual loss-of Rac1 and Rac1b functions in MMTV-NIC mouse model delays tumor latency in a dose-dependent manner (Fig. 1E). To determine whether the loss-of Rac1b is responsible for this phenotype, we analyzed the impact of Rac1b deficiency alone on palpable tumor formation. Our results revealed similar tumor latencies for Rac1b^−/−^;MMTV-NIC, Rac1b^+/−^;MMTV-NIC and Rac1b^+/+^;MMTV-NIC mice (Fig. 6A), indicating that the tumor latency phenotype observed in Rac1^flox/flox^;MMTV-NIC and Rac1^flox/+^; MMTV-NIC mice is due to the loss of Rac1, not Rac1b.

Since the loss-of RAC1B in MCF7 cells results in BCSC depletion in vivo (Fig. 4D), we examined whether Rac1b is also required for BCSCs in the MMTV-NIC mouse model. We performed mammosphere assay using CD49f^+^CD24^+^ tumor cells isolated from Rac1b^+/+^;MMTV-NIC (or Rac1b^+/−^;MMTV-NIC) and Rac1b^−/−^;MMTV-NIC tumors (Fig. 6B, C). Our results revealed a 65% decrease in mammosphere-forming BCSC frequency in Rac1b-null tumors compared with Rac1b-proficient tumors, suggesting that Rac1b may be crucial for BCSC self-renewal/maintenance.

To better identify the Rac1b-expressing cells within MMTV-NIC tumors, we generated a new transgenic mouse line, Rac1b^RFP/+^, by utilizing CRISPR-targeting approach coupled with homology-directed repair (HDR) template to knock-in a T2A-mRFP cassette in-frame within the exon3b of *Rac1* gene. The choice of HDR template was experimentally optimized by using the murine mammary epithelial cell line, EPH4, to ensure achieving a successful knock-in without disrupting proper splicing of the transgenic mRNA (Supplementary Fig. 7A–D). RT-PCR analysis of RFP^+^ and RFP^-^ cells sorted from the mammary glands of nulliparous Rac1b^RFP/+^ mice confirmed that mRFP expression in this mouse line can serve as a surrogate reporter for Rac1b splicing (Supplementary Fig. 7E, F).

**Fig. 7:**
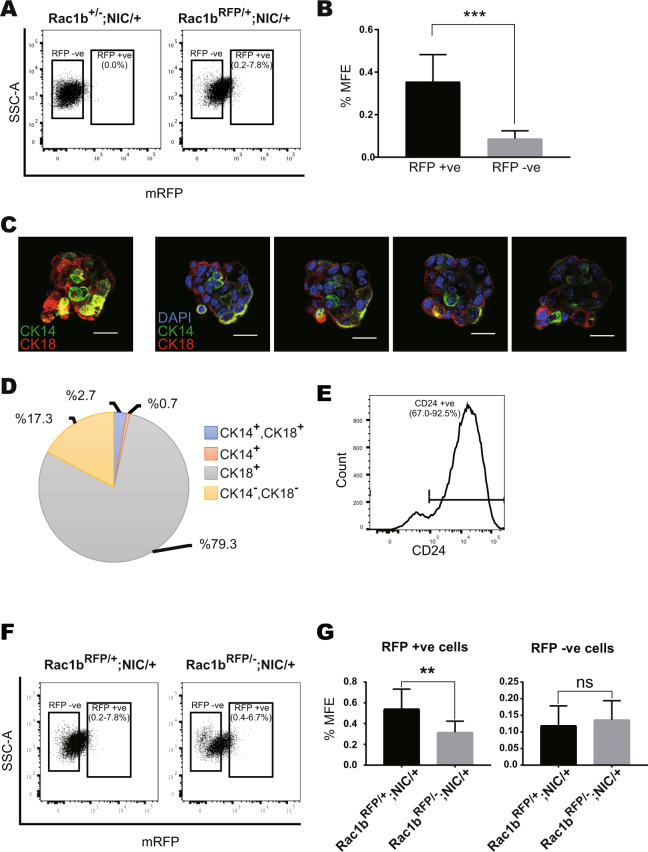
Rac1b function is required for the maintenance of a large subset of BCSCs expressing Rac1b. **A** Representative dot plots for flow cytometry analysis of primary mammary tumor cells from Rac1b^+/−^;MMTV-NIC and Rac1b^RFP/+^;MMTV-NIC mice are shown for the single, alive, lineage-negative cell population. The observed range for RFP^+^ cell frequency in tumors of different animals is shown within the RFP^+^ gate (*n* = 6). **B** Mammosphere forming efficiency (%MFE) of RFP^+^ versus RFP^-^ cells sorted from Rac1b^RFP/+^;MMTV-NIC tumors as shown in (**A**). Values represent the mean ± SEM of 6 animals. (***p < 0.005; paired *t*-test). **C** Representative confocal microscopy images of a primary mammosphere formed by Lin^-^RFP^+^ tumor cells sorted from Rac1b^RFP/+^;MMTV-NIC mammary tumors (*n* = 3 mice). The mammosphere was coimmunostained for CK18 and CK14 expression. The leftmost image shows the deconvoluted image for CK14 (green) and CK18(red). Individual images of confocal planes selected to represent different Z-stack positions are shown together with DAPI-staining (shown in blue) serving as a nuclear stain. Scale bars represent 20 um. **D** Pie chart representation of the distribution of CK-18 and/or CK-14 expressing Lin^-^RFP^+^ tumor cells sorted from Rac1b^RFP/+^;MMTV-NIC breast tumors. Values represent the mean data for 3 independent tumors analyzed. **E** Representative histogram of flow cytometry analysis for CD24 expression in Lin^-^RFP^+^ cells sorted from Rac1b^RFP/+^;MMTV-NIC tumors. The observed range for the CD24^+^ cell frequency in tumors of different animals is shown on the histogram (*n* = 3). **F** Representative dot plots of flow cytometry analysis for primary mammary tumor cells of Rac1b^RFP/+^;MMTV-NIC and Rac1b^RFP/−^;MMTV-NIC mice are shown for the single, alive, lineage-negative cell population. The observed range of RFP^+^ cell frequency in tumors of different animals with same genotype is shown within the RFP^+^ gate (*n* = 9). **G** Mammosphere forming efficiency (%MFE) of RFP^+^ and RFP^-^ cells sorted from Rac1b^RFP/+^;MMTV-NIC and Rac1b^RFP/−^;MMTV-NIC tumors as shown in (**F**). Values represent the mean ± SEM of 3 animals. (**p < 0.01, ns not significant; paired *t*-test).

We then generated the Rac1b^RFP/+^;MMTV-NIC mice to analyze whether Rac1b is expressed by BCSCs in Neu-driven tumors. The RFP^+^ (i.e., Rac1b-expressing) cells in these tumors constituted a small population of lineage (CD31, CD45, TER119)-negative cells (Fig. 7A), which displayed a 4-fold enriched frequency of mammosphere-forming cells compared with the Lin^-^RFP^-^ population (Fig. 7B and Supplementary Fig. 8A). Immunostaining of Lin^-^RFP^+^ tumor cell-driven primary mammospheres revealed that most of these mammospheres (~90%) were composed of cells expressing CK18 luminal and/or CK14 basal epithelial lineage markers (Fig. 7C). Given that not all RFP^+^ cells were mammosphere-forming BCSCs, we further investigated the composition of Rac1b-expressing cell populations in these tumors by immunostaining the sorted Lin^-^RFP^+^ cells for CK18 and CK14 (Supplementary Fig. 8B). Our results revealed that an average of 79.3% of Lin^-^RFP^+^ cells were expressing CK18, whereas 2.7% were positive for both CK14 and CK18 (Fig. 7D). Furthermore, the flow cytometry analysis showed that an average of 84% of the Lin^-^RFP^+^ cells from Rac1b^RFP/+^;MMTV-NIC tumors were also CD24^+^ (Fig. 7E). Together, these results indicate that in MMTV-NIC tumors Rac1b is expressed in a small population of tumor epithelia that also contains a substantial subset of BCSCs.

Next, we analyzed Rac1b-proficient (Rac1b^RFP/+^;MMTV-NIC) and Rac1b-null (Rac1b^RFP/−^;MMTV-NIC) tumors to determine whether the decrease in BCSC frequency observed in Rac1b^−/−^;MMTV-NIC tumors is due to a change in RFP^+^ BCSCs. In both genotypes, Lin^-^RFP^+^ cells formed a similar size subpopulation (Fig. 7F). However, there were approximately 42% fewer mammosphere-forming BCSCs in the Lin^-^RFP^+^ population of Rac1b-null tumors compared with the same population in Rac1b-proficient tumors (Fig. 7G). In contrast, mammosphere-forming efficiency of Lin^-^RFP^-^ cells did not show a significant difference between genotypes.

Collectively, these results demonstrate that Rac1b is expressed in a substantial subset of BCSCs, which require Rac1b function for their maintenance in vivo.

### Loss of Rac1b increases the chemosensitivity of primary breast tumor cells

**Fig. 8:**
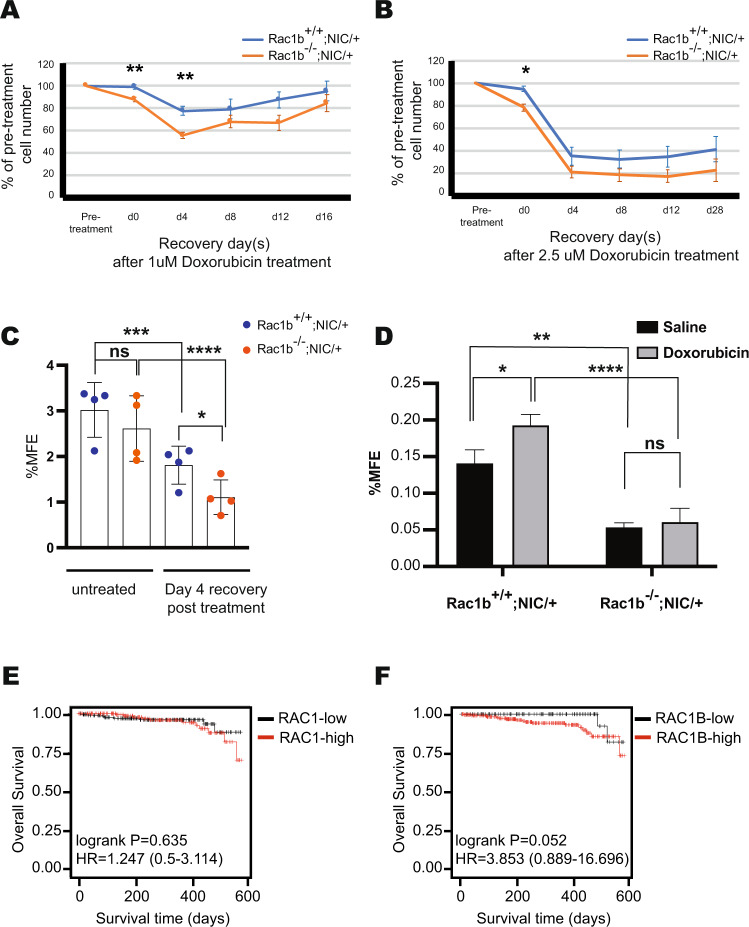
Rac1b expression determines the chemosensitivity of breast tumors to doxorubicin treatment in vitro, in vivo and in patients. **A**, **B** Cell growth curve of primary tumor cell lines obtained from Rac1b^+/+^;MMTV-NIC (*n* = 4) and Rac1b^−/−^;MMTV-NIC (*n* = 4) mammary tumors in post-treatment recovery period after 1 uM (**A**) or 2.5 uM (**B**) doxorubicin treatment for 24 h. Cell numbers are normalized to the pre-treatment cell number for each primary cell line and percentage changes are represented as the mean ± SEM of 4 independent lines for each genotype with averages taken from 3 independent experiments. (*p < 0.05 and **p < 0.01; two-tailed unpaired *t*-test). **C** Mammosphere forming efficiency (%MFE) of Rac1b^+/+^;MMTV-NIC (*n* = 4) and Rac1b^−/−^;MMTV-NIC (*n* = 4) primary tumor cell lines either in the absence of doxorubicin treatment or 4 days after 24 h treatment with 2.5 uM doxorubicin. Values represent the mean ± SD of 4 primary cell lines. Each data point shown represents the average of 3 independent experiments for each individual primary cell line. (*p < 0.05, ***p < 0.0005, ****p < 0.0001; two-way-ANOVA). **D** Mammosphere forming efficiency (%MFE) of sorted tumor cells obtained from the Rac1b^+/+^;MMTV-NIC and Rac1b^−/−^;MMTV-NIC mice treated in vivo with saline as control or with Doxorubicin (10 mg/kg body weight) on the day after the second cycle of treatment. Values represent the mean ± SEM of *n* = 4 saline-treatment and *n* = 5 for doxorubicin-treatment groups for each genotype. (*p < 0.05, **p < 0.005, ****p < 0.0001; two-way-ANOVA). **E**, **F** Kaplan–Meier plots showing overall survival probability in doxorubicin/adriamycin-treated patients with breast cancer obtained from the TCGA dataset (*n* = 361) and stratified by RAC1 (**E**) or RAC1B (**F**) expression in their tumors. Expression levels and survival data were retrieved from TSVdb. Hazard ratio (HR) and respective confidence intervals are shown. P values are determined by Log Rank test.

RAC1B function is required for the chemoresistance of MCF7 cells to doxorubicin treatment (Fig. 3) and for the BCSC maintenance in Neu-driven tumors in mice (Fig. 7). We therefore investigated whether Rac1b also affects chemoresistance in Neu-driven tumors by treating the primary cell lines, which we have generated from Rac1b^+/+^;MMTV-NIC and Rac1b^−/−^;MMTV-NIC tumors, with either 1 uM or 2.5 uM doxorubicin for 24 h. The relative cell loss was significantly higher in Rac1b-null lines compared with Rac1b-proficient lines in both doxorubicin-treatment groups (Fig. 8A, B; Day 0 samples), demonstrating an increased cytotoxic response of Rac1b-null tumor cells to doxorubicin.

The sustained cytotoxic effect of doxorubicin during the initial 4 days after the removal of the chemotherapeutic agent was observed in both genotypes. However, there was a significantly higher cell loss in Rac1b-null lines treated with 1uM doxorubicin (Fig. 8A, B and Supplementary Fig. 9; Day 4 samples). We have observed that 3 out of 4 Rac1b-null and 1 out of 4 Rac1b-proficient primary cell lines showed no cell growth throughout the whole 28 days recovery period after 2.5 uM doxorubicin treatment (Supplementary Fig. 9). These differences between the primary cell lines of the same genotype groups may reflect the inter-tumor heterogeneity, as they had been derived from tumors of different animals, which would have acquired different sets of mutations during the process of tumorigenesis. Nevertheless, our results demonstrate that doxorubicin treatment achieves a higher level of cytotoxic effect in Rac1b-null tumor cells, which are three times more likely to show no recovery after the 2.5 uM doxorubicin treatment.

To determine whether this inter-tumor heterogeneity could be explained by altered expression levels of Rac1 and/or Rac1b in these primary cell lines, we have performed qRT-PCR analysis (Supplementary Fig. 10A). The chemosensitive Rac1b-proficient primary cell line had similar levels of Rac1 and Rac1b transcripts as the other 3 chemoresistant lines, indicating that the observed difference in chemosensitivity is not due to an alteration in Rac1b splicing. However, we have observed a slight, but not significant, upregulation of Rac1 transcript levels in chemoresistant Rac1b-null primary cell line, which may suggest a potential compensation for the loss-of Rac1b function by increased Rac1 expression.

Next, we performed mammosphere assay to elucidate the effects of doxorubicin treatment on the BCSC population in these primary cell lines (Fig. 8C and Supplementary Fig. 10B). Both Rac1b-null and Rac1b-proficient primary cell lines had similar mammosphere-forming efficiency in the absence of doxorubicin treatment. However, there were significantly less mammosphere-forming cells in Rac1b-null cell lines compared with Rac1b-proficient cell lines when analyzed four days after the 24-h-long 2.5 uM doxorubicin exposure. Although doxorubicin treatment led to a significant reduction in the BCSC population of both Rac1b-null and Rac1b-proficient cell lines, a higher level of reduction was observed for Rac1b-null cell lines, suggesting that Rac1b function is essential for the chemoresistance of BCSCs to doxorubicin treatment.

To confirm the in vivo relevance of these findings obtained from primary cell lines, we have treated Rac1b^+/+^;MMTV-NIC and Rac1b^−/−^;MMTV-NIC mice with 2 cycles of doxorubicin (10 mg/kg body weight) or saline at 3- and 4-weeks after they have developed palpable tumors. The day after their second cycle of treatment, tumors were dissected to isolate CD49f^+^;CD24^+^ tumor epithelia by FACS (Supplementary Fig. 11) and plated in mammosphere culture. In saline-treated control groups, tumors of the Rac1b^−/−^;NIC/ + mice contained a significantly lower frequency of BCSCs compared to those of Rac1b^+/+^;NIC/ + mice (Fig. 8D), confirming our previous findings in tumors of untreated mice (Fig. 6C). Compared with the saline treated controls, doxorubicin treatment led to a significant increase of the BCSC frequency only in Rac1b^+/+^;NIC/ + , but not in Rac1b^−/−^;NIC/ + tumors (Fig. 8D). As the doxorubicin treatment of tumor-bearing mice led to shrinking of their tumors to an almost unpalpable size, an increase in BCSC frequency in Rac1b-proficient tumors of doxorubicin-treated mice may reflect a higher survival of BCSCs compared to those in Rac1b-null tumors.

Taken together, our results for doxorubicin treatment on primary cell lines in vitro *and* tumor-bearing mice in vivo suggest that Rac1b function is crucial for the doxorubicin-resistance of BCSC populations in Neu-driven tumors.

### RAC1B levels are predictive of overall survival in response to doxorubicin treatment in patients with breast cancer

After establishing in experimental models of breast cancer that RAC1B function is essential for the chemosensitivity of breast tumor cells, and in particular BCSCs, to the effects of doxorubicin treatment, we have sought to determine the clinical relevance of our findings. Thus, we have analyzed the RNAseq dataset within the TCGA database for breast cancer using the TSVdb annotations of spliced variants [45] (Supplementary Fig. 12). Our results demonstrated an inverse correlation of RAC1 and RAC1B expression in terms of the ER, PR, or HER2 status of breast tumors. High RAC1 transcript levels were significantly correlated with tumors that were ER-negative, PR-negative, or HER2-positive. In contrast, higher expression of RAC1B was significantly correlated with ER-positive, PR-positive, HER2-negative or HER2-equivocal tumors. Neither RAC1 nor RAC1B transcript levels were predictive of overall survival when all patients were considered. However, the expression levels of RAC1B, but not RAC1, was predictive of overall survival in doxorubicin-treated patient cohort, with higher RAC1B expression resulting in worse prognosis with a hazards ratio of 3.853 (Fig. 8E, F).

These observations confirm our findings in experimental models of breast cancer suggesting that RAC1B may function to ensure the chemoresistance of breast tumors to the effects of doxorubicin treatment. Thus, RAC1B can be a clinically relevant molecular target, as its therapeutic inhibition may improve the success of doxorubicin chemotherapy in patients with breast cancer.

## Discussion

Developing CSC-targeting treatments to ablate tumors at their root has been a research aim for over two decades. However, this idea has proven elusive in breast cancer due to high levels of heterogeneity within the BCSC pool and the lack of appropriate molecular targets showing tumor-specific stem cell selectivity for their functional indispensability. Here, we have demonstrated that RAC1B, the alternatively spliced variant of RAC1, is required for the BCSC plasticity, self-renewal and chemosensitivity to doxorubicin treatment. Given that the loss-of Rac1b function does not lead to any apparent developmental or physiological health problems in vivo and that Rac1b is dispensable for MaSCs and normal mammary gland development/function, variant-specific targeting of RAC1B can be a clinically relevant option to developing novel BCSC-targeted therapies.

Previous studies using xenograft models showed that RAC-inhibition via EHT-1864 or EHop-016 can delay tumor growth and metastasis of breast tumor cells in vivo [46, 47]. As overall RAC inhibition can lead to BCSC depletion (Fig. 1), the beneficial effects seen in these studies could in part be attributed to the indispensable function of RAC signaling in BCSCs. However, these studies also reported that the blood serum concentrations of these inhibitors need to be low, as higher doses showed toxic effects in mice. This is unsurprising as Rac1, unlike Rac1b, is indispensable for normal development and physiology [17–20].

In addition to MCF7, we also generated RAC1B-null single-cell clones of human breast cancer cell lines T47D and SKBR3 to analyze the loss-of RAC1B function phenotypes in their chemosensitivity to doxorubicin treatment. However, as the parental cell lines and the RAC1B-proficient single-cell clones were already highly sensitive to doxorubicin treatment (i.e no obvious recovery of cell growth post-treatment), the loss-of RAC1B function did not provide any additional benefit in increasing their chemosensitivity (Supplementary Fig. 13). Interestingly, the analysis of a previously published exon array dataset for 40 different breast cancer cell lines [48] revealed that MCF7 has higher RAC1B transcript levels compared with most other cell lines, including T47D and SKBR3. This may indicate a correlation between RAC1B transcript levels and the chemosensitivity to doxorubicin treatment in breast cancer cell lines, although this hypothesis needs to be experimentally verified in future studies. Furthermore, we showed that high RAC1B transcript levels can be predictive of worse overall survival in patients treated with doxorubicin (Fig. 8F). As patients with high tumor levels of RAC1B could potentially present chemoresistance and/or early tumor relapse, this patient subgroup would be most likely to benefit from a variant-specific targeting of RAC1B in a combination treatment with doxorubicin.

## Materials and methods

### Mouse experiments

All mouse experiments were conducted under license in accordance with the UK Home Office Animals (Scientific Procedures) Act (1986) regulations with the approval of study protocols by the Animal Welfare and Ethical Review Body (AWERB) of the University of Manchester. Mice were maintained in a pathogen-free facility at the University of Manchester and kept in 12-h light-dark cycles in temperature- and humidity-controlled environment and were provided with food and water ad libitum.

The mouse lines Rac1^flox^ and MMTV-NIC were previously described [32, 33]. Rac1b^−/−^ mouse line was generated by crossing the Rac1b^flox/flox^ mice [16] with a universal CRE-deleter mouse line [49] and subsequently breeding out the CRE-transgene and backcrossing into C57BL6/J and FVB/J backgrounds for at least 7 generations. Rac1b^RFP/+^ mouse line in pure FVB/J background has been generated in this study using HDR-coupled CRISPR-targeting as described in Supplementary Methods. Mice at defined pregnancy stages were obtained by timed mating with the morning of vaginal plug observation being considered as 0.5 days-post-coitum (d.p.c). Whole-mount staining of No4 mammary glands were performed as described elsewhere [50].

Xenograft transplantations of MCF7 cells were performed as previously described [51]. Briefly, adult nude mice (Charles River) were injected with 100,000 cells sub-cutaneously into both right and left ventral flanks and provided with estrogen (E2, SIGMA) in drinking water at a concentration of 8 mg/ml throughout the experiment. Tumor growth was monitored with routine measurements using a caliper.

### Statistical analysis

Statistical tests used in this study were selected based on population distribution, data type and sample centrality/variability to meet assumptions of tests using GraphPad Prism and Microsoft Excel software. *P* < 0.05 were considered statistically significant. Data are expressed as mean ± standard deviation (SD) or mean ± standard error of the mean (SEM).

All other methods and statistical approaches used in this study are described in the Supplementary methods.

## Supplementary information

Supplementary Material

## Data Availability

All relevant data are available from the authors upon request.
