# Supplementary material for: RAC1B function is essential for breast cancer stem cell maintenance and chemoresistance of breast tumor cells
Source: Oncogene. 2023 Jan 5;42(9):679–92. doi: 10.1038/s41388-022-02574-6 (PMC9957727; doi:10.1038/s41388-022-02574-6)
Supplement: Supplementary file 1 — Supplementary Material [file 41388_2022_2574_MOESM1_ESM.pdf]

Supplementary Information for:

RAC1B function is essential for breast cancer stem cell maintenance and chemoresistance of breast tumour cells

Fuhui Chen, Sevim B. Gurler, David Novo, Cigdem Selli, Denis G. Alferez, Secil Eroglu, Kyriaki Pavlou, Jingwei Zhang, Andrew H. Sims, Neil E. Humphreys, Antony Adamson, Andrew Campbell, Owen J. Sansom, Cathy Tournier, Robert B. Clarke, Keith Brennan, Charles H. Streuli, Ahmet Ucar

Supplementary Materials and Methods
Supplementary References
Figures S1 to S13

Supplementary Materials and Methods

Cell lines and Culture

MCF7, T47D, BT474, SKBR3, MDA-MB231, and EPH4 cell lines were purchased from American Tissue Culture Collection (ATCC); JIMT-1 cell line was purchased from German Collection of Microorganisms and Cell cultures (DSMZ). Cell lines were routinely tested for mycoplasma contamination and were mycoplasma-free. STR profiling of parentall cell lines were performed in every 10 passages. They were cultured in adherence with DMEM (Sigma Aldrich) supplemented with 10% FBS (LabTech). EHT-1864 and EHOP-016 inhibitors were purchased from Stratech Scientific. Doxorubicin and doxycycline were purchased from Merck.

Primary tumour cell lines were obtained by culturing FACS sorted cells in adherent cultures with DMEM/F12 medium (Sigma Aldrich) supplemented with 10% FBS, 5µg/ml Insulin (#I9278, Sigma Aldrich), 0.02µg/ml EGF (#E4127, Sigma Aldrich), and 1% Penicillin-Streptomycin (Sigma Aldrich).

Plasmids and generation of transgenic cell lines

CRISPR/Double nickase targeting in cell lines were performed using the protocols described in [1]. For targeting human *RAC1B*, CRISPR targeting constructs were prepared using the pSpCas9n(BB)-2A-GFP (pX461, Addgene #48140) vector to express sgRNA-hA (CCUAGUAAUGCAUGAGAACU) and sgRNA-hB (ACGGUAAGGAUAUAACCUCC). For targeting mouse *Rac1b*, the same vector backbone was used to generate constructs to express sgRNA-mA (UGUCUCCAACCUAGUAAUGC) and sg-RNA-mB (GGUAAAGAUAGACCCUCCAG) and HDR templates were cloned into pGEM-T vector backbone. Co-transfected cells (i.e. GFP^+) were sorted 24 hours after transfection directly into the 96 well plates as one cell per well using FACS (ARIA Fusion, BD Biosciences) and grown as single-cell clones.

For generating stable transgenic MCF7 cells for doxycycline-inducible RAC1B expression, we used the pCDH lentiviral backbone and cloned the coding sequence for RFP-RAC1B (human) fusion protein downstream of TREt promoter. Lentiviral transduction of MCF7 cells was performed as previously described [2] and transduced cells were FACS sorted based on mRFP expression.

Generation of Rac1b^{RFP} mouse line by CRISPR-Cas9:

CRISPR sgRNA sequences targeted proximally to the insert site (TGTCTCCAACCTAGTAATGC and GGTAAAGATAGACCCTCCAG) were purchased as crRNA oligos and annealed with tracrRNA (both oligos supplied by Integrated DNA Technologies) in sterile, RNase free injection buffer (TrisHCl 1mM, pH 7.5, EDTA 0.1mM) by combining 2.5 ug crRNA with 5 ug tracrRNA and heating to 95oC, which was allowed to slowly cool to room temperature. For our donor repair template, we used the EASI-CRISPR long-ssDNA strategy [3], and generated a homology flanked lssDNA donor using protocols described in [4].

For embryo microinjection the annealed sgRNA was complexed with Cas9 protein (New England Biolabs) at room temperature for 10 minutes, before addition of long ssDNA donor (final concentrations; sgRNA 20 ng/ul, Cas9 protein 20 ng/ul, lssDNA 10 ng/ul). CRISPR reagents were directly microinjected into FVB (Envigo) zygote pronuclei using standard protocols. Zygotes were cultured overnight and single-cell embryos surgically implanted into the oviduct of day 0.5 post-coitum pseudopregnant mice.

Potential founder mice were screened by PCR, first using primers that flank the sgRNA sites (Frw: tccagctgcatggagtaatg, Rev: gcagcctctcctgagtgagt), which both identifies editing activity in the form of InDels from NHEJ repair in almost every pup and can also detect larger products implying HDR. Secondary PCRs used the same primers in combination with mRFP1 primers (mRFP1_R: cgccctcgatctcgaact, mRFP1_F: ccgaggtcaagaccacctac). A founder giving positive products in all three PCR reactions was further characterised by Sanger sequencing of the PCR amplicons of the initial PCR.

RT-PCR analysis

Isolated total RNA samples were reverse-transcribed by using Tetro cDNA Synthesis Kit (#BIO-65043, Bioline) using random-hexamer primers. The cDNA products were then amplified by using gene-specific primers and GoTaq DNA polymerase (#M7845, Promega) on Veriti Thermal Cycler (Applied Biosystems). For qRT-PCR, TaqMan assays were used for Rac1 (Mm01201653_mH), HPRT (Mm03024075) and Rac1b (Custom design primers: 5′-TGGACAAGAAGATTATGACAGATTGC-3′ and 5′-CCCTGGAGGGTCTATCTTTACCA-3′, with a probe 5′-CCGCAGACAGTTGGAGA-3′).

Immunoblot analysis

Immunoblot experiments were performed as previously described [2]. The primary antibodies used in this study were anti-mouse Rac1 (#23A8, Merck), anti-human Rac1 (#05-389, Millipore), anti-human Rac1b (#09-271, Millipore) and anti-β-actin (#8229, Abcam). Secondary antibodies used were IRDye-800CW donkey anti-mouse and IRDye-680RD donkey anti-goat. The membranes were visualised and analysed using Odyssey CLx Imaging System and Image Studio software (LI-COR).

Primary cell isolation

Dissected breast tumours or mammary glands from mice were minced into small pieces and digested in 1 mg/ml Collagenase (#11088793001, Roche) and 0.08 mg/ml Hyaluronidase (#H3506, Sigma) digestion cocktail at 37℃ for about 4 hours. After dissociation, the reaction was stopped by diluting in HBSS (#H9394, Sigma) with 2% FBS, and tissue pellets were washed with pure HBSS. Samples were then digested using Trypsin-EDTA (Sigma), followed by digestion in 1 mg/ml Dispase II (#4942078001, Roche) and 0.4 mg/ml DNase I (#11284932001, Roche) to obtain single cell suspensions.

Flow cytometry analysis and FACS sorting

All flow cytometry experiments were performed using either LSR Fortessa or ARIA Fusion (Becton Dickinson(BD)). For cell line studies, cells were collected with trypsinisation and stained with ALDEFLUOR staining kit (Stem Cell Technologies) or co-stained with CD24-APC-H7 (#658331, BD) and CD44-APC (#559942, BD) using manufacturer's protocols. For staining human cells recovered from explants, CD298-APC (Biolegends) was used along with propidium iodide (Sigma Aldrich).

Primary murine cells isolated from mammary glands or breast tumours were blocked with CD16/CD32 (#553141, BD) and stained with CD45-Biotin (#553077, BD), CD31-Biotin (#558737, BD), Ter119-Biotin (#553672, BD), CD24-APC (#562349, BD), CD49f-eFluor450 (#48-0495-92, Invitrogen), Streptavidin-PE-Cy7 (#557598, BD), or Streptavidin-FITC (#554060, BD) antibodies. The Dead/live cell markers used were either DRAQ7 (Biostatus) or DAPI (Sigma).

Mammosphere-formation assay:

Single cells were plated in 8 technical replicates within 96 well ultra-low attachment plates in sphere-forming medium as described in [5]. For cell lines, cells were plated at a concentration of 1 000 cells/ml and 10 000 cells/ml. For cells sorted from mammary glands, they were plated at a concentration of 10 000 cells/ml and 100 000 cells/ml. Primary tumor cells were plated at a concentration of 50 000 cells/ml and 100 000 cell/ml. Mammosphere numbers were quantified at Day 5 or Day 7 of mammosphere culture of cell lines or primary cells, respectively. Solid structures with a minimum diameter of 50 um on two perpendicular axes are considered as mammospheres. For calculations of mammosphere forming efficiency, the values obtained from the lowest cell concentration that provides at least 1 sphere per well has been used to avoid the bias from the merging of developing spheres at higher concentrations.

Doxorubicin treatment

Cells were seeded in flat-bottom 96-well plates. Once they reach >90% confluence, they were treated with doxorubicin (#BP990, Sigma) for 24 or 48 hours and then washed several times to remove the chemotherapeutic agent. For cell quantification, AlamarBlue reagent (#799771, Invitrogen) was used according to the manufacturer's protocols and absorbances of reduced versus oxidised reagents were measured at 562 nm and 630 nm wavelengths using plate reader ELx800 (BioTek).

For doxorubicin treatment *in vivo*, tumor-bearing transgenic mice were injected with saline (i.e. vehicle-only control group) or doxorubicin (10 mg/kg body weight) via intraperitoneal route 20 days after they have developed palpable tumours. A second cycle of saline or doxorubicin injection was given 7 days after the first cycle and animals were culled 24 hours after the second dosing for collection of tumors for further analysis.

Immunofluorescence

Immunofluorescence for cytospinned cells:

Sorted primary cells were fixed in 4% paraformaldehyde solution and then immobilised onto Poly-lysine slides (ThermoFisher J2800AMNZ) using cyto-spin at a concentration of 10 000 cells per slide. Samples were blocked with 0.2% Triton and 5% horse serum in PBS prior to incubation with primary antibodies against CK14 and CK18 in room temperature for 1 hour.

After a subsequent incubation with secondary antibodies and DAPI for 1 h at room temperature in dark, they were mounted with Fluorescence mounting medium (#S3023, DAKO). Stained samples were pictured using Zeiss Imager M2 fluorescent microscope with 63x objective.

Immunofluorescence for 3D spheres derived from mammosphere assay:

The organoids formed in mammosphere culture were collected and fixed in 4% paraformaldehyde solution for 20 min. They were then blocked and permeabilized with organoid washing buffer (0.1% Triton-X100, 5% BSA in 1xPBS) for 15 min, followed by incubation with primary antibodies against CK14 and CK18 for 1 h at room temperature. After 3 rounds of washing with organoid washing buffer, the organoids were then incubated with secondary antibodies and DAPI for 1 h at room temperature in dark. Organoids were then transferred into 6 well plate containing 1xPBS and imaged by using Leica SP8 Upright dipping lens Confocal microscope with 63x objective.

The primary antibodies used in the experiments described above were cytokeratin 14 Polyclonal Antibody (#PA5-16722, Invitrogen) and anti-Keratin K18 Mouse Monoclonal Antibody (#61028, Progen), used in 1:100 dilution. The secondary antibodies used were donkey anti-Rabbit IgG (H+L) Alexa Fluor 488 (#21206, Invitrogen) and donkey anti-Mouse IgG (H+L) Alexa Fluor 594 (#21203, Invitrogen), used in 1:500 dilution. DAPI was used at 0.1µg/ml concentration.

Statistical analysis:

Tumor latency analysis

All mice bearing an MMTV-NIC allele has been checked for palpable tumour formation twice weekly after they reach the age of 80 days-old. The age when the palpable tumors were observed has been recorded. The minimum sample size, which was chosen by performing a power analysis to determine with confidence +/- 20% meaningful difference with p=0.05 and 80% power, was n=13. No animals were excluded from the analyses during the study. The investigator recording the ages of mice with palpable tumor formation was blinded to the genotypes of animals.

In vivo doxorubicin treatment

Animals were randomized for treatment options independent of their age when they have formed palpable tumors. The investigator performing the injections were blinded to the

genotype and the content of injected material. The sample size was initially chosen arbitrarily and evaluated retrospectively using the data on the variability of response in Rac1b$^{+/+}$;MMTV-NIC mice. The power analysis has revealed a sample size of n=4 is required for determining a meaningful difference with +/- 20 confidence with p=0.05 and 80% power. No animals were excluded from the analyses during the study.

Data with high levels of variation

For experiments, in which there was higher levels of variation, individual data points have been shown along with bar or line graphs representing mean values.

TCGA dataset analysis:

R and Bioconductor [6] were used for statistical analysis and data visualisation. TCGA isoform expression and survival data of breast cancer samples were retrieved from TSVdb [7]. Breast cancer molecular subtypes of TCGA samples and treatment information were retrieved from TCGABiolinks [8]. Gene expression was given as log2 transformed normalized RSEM. The association between expression and overall survival in TCGA breast cancer patients was determined using a univariate Cox proportional regression model. survivALL package (https://github.com/pearcedom/survivALL) was used for dichotomising in survival analysis. All possible cut-offs for dichotomising samples were analysed. Two-tailed student t test and ANOVA with post-hoc Tukey HSD for multiple comparison were used to calculate statistical significance. Differences were considered significant at P < 0.05. Data was visualized using ggplot2 (https://ggplot2.tidyverse.org) and GGally (https://ggobi.github.io/ggally)libraries.

Supplementary References:

1 Ran FA, Hsu PD, Wright J, Agarwala V, Scott DA, Zhang F. Genome engineering using the CRISPR-Cas9 system. *Nat Protoc* 2013; 8: 2281-2308.

2 Xu Q, Zhang J, Telfer BA, Zhang H, Ali N, Chen F *et al*. The extracellular-regulated protein kinase 5 (ERK5) enhances metastatic burden in triple-negative breast cancer through focal adhesion protein kinase (FAK)-mediated regulation of cell adhesion. *Oncogene* 2021; 40: 3929-3941.

3 Quadros RM, Miura H, Harms DW, Akatsuka H, Sato T, Aida T *et al*. Easi-CRISPR: a robust method for one-step generation of mice carrying conditional and insertion alleles using long ssDNA donors and CRISPR ribonucleoproteins. *Genome Biol* 2017; 18: 92.

4 Bennett H, Aguilar-Martinez E, Adamson AD. CRISPR-mediated knock-in in the mouse embryo using long single stranded DNA donors synthesised by biotinylated PCR. *Methods* 2021; 191: 3-14.

5 Ucar A, Ucar O, Klug P, Matt S, Brunk F, Hofmann TG *et al*. Adult thymus contains FoxN1(-) epithelial stem cells that are bipotent for medullary and cortical thymic epithelial lineages. *Immunity* 2014; 41: 257-269.

6 Gentleman RC, Carey VJ, Bates DM, Bolstad B, Dettling M, Dudoit S *et al*. *Genome Biology* 2004; 5.

7 Sun W, Duan T, Ye P, Chen K, Zhang G, Lai M *et al*. TSVdb: a web-tool for TCGA splicing variants analysis. *BMC Genomics* 2018; 19.

8 Colaprico A, Silva TC, Olsen C, Garofano L, Cava C, Garolini D *et al*. TCGAbiolinks: an R/Bioconductor package for integrative analysis of TCGA data. *Nucleic Acids Res* 2016; 44: e71.

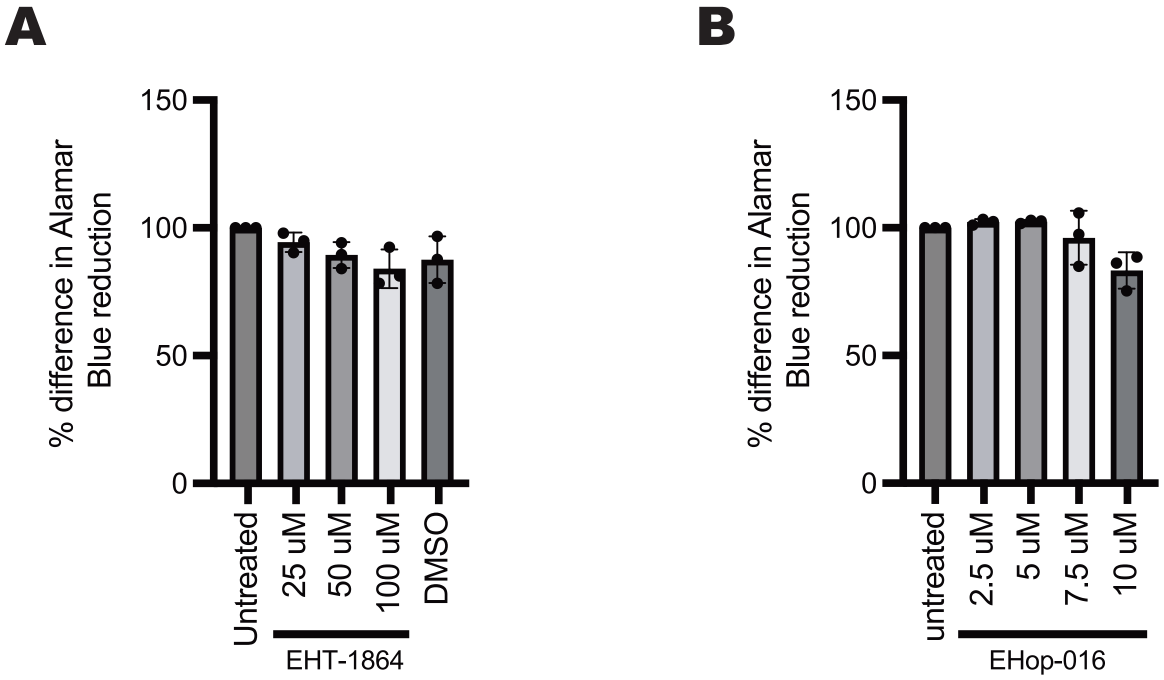

Supplementary Figure 1. Inhibition of RAC signaling does not cause overall cytotoxicity in MCF7 cells

A,**B**) MCF7 cells were treated with RAC inhibitors EHT1864 (**A**) or EHop-016 (**B**) in monolayer cultures with the indicated concentrations for 24 hours followed by quantification of cell numbers using Alamar-Blue staining. Percent reduction of AlamarBlue values for treatment groups were normalized to the levels of corresponding untreated groups. Values represent the mean ± SD of 3 independent experiments and the data for each experiment is shown as individual data points. DMSO group in (**A**) is the vehicle-only control and corresponds to the same concentration of DMSO as in the 100 uM EHT-1864-treated group. No significant difference was observed in any treatment groups compared to untreated MCF7 cells using one-way ANOVA test.

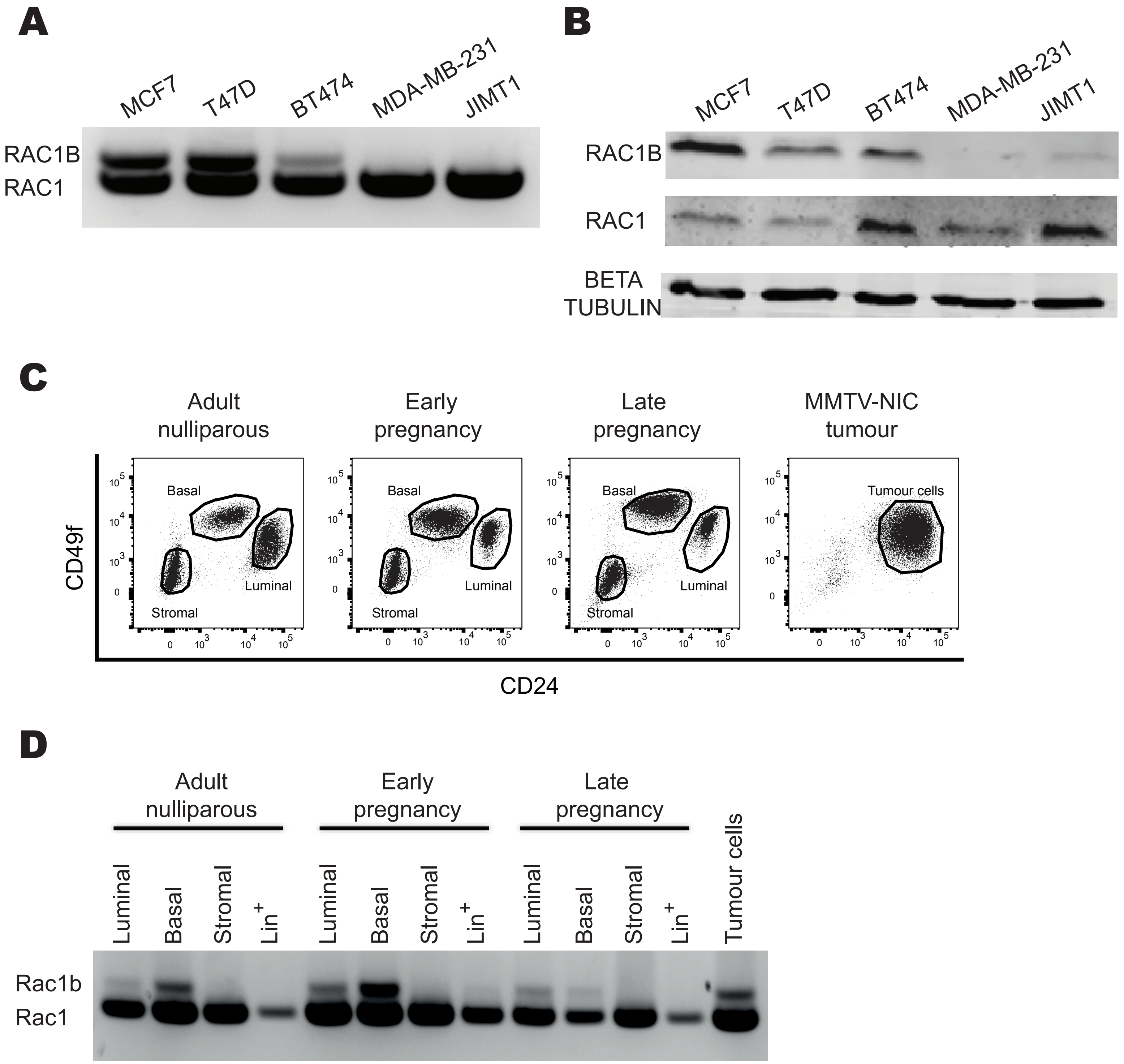

Supplementary Figure 2. RAC1 and RAC1B expression in human breast cancer cell lines, murine mammary gland and breast tumours.

A, B) RT-PCR (**A**) and immunoblot analysis (**B**) of RAC1 and RAC1B expression in human breast cancer cell lines representing different breast cancer subtypes. Beta-tubulin was used as a loading control in immunoblot experiments.

C) Dot plots of CD49f versus CD24 expression that was used for sorting basal, luminal and stromal subpopulations within the alive, single, and lineage (CD45, TER119, CD31)-negative cell population obtained from mammary glands of mice in adult nulliparous, early-pregnancy (12 d.p.c) and late-pregnancy (18 d.p.c) stages; and for sorting tumour cells within the alive, single, and lineage-negative cell population obtained from MMTV-NIC breast tumours.

D) RT-PCR analysis of Rac1 and Rac1b expression in sorted cell populations from murine mammary glands and breast tumours as shown in (**C**).

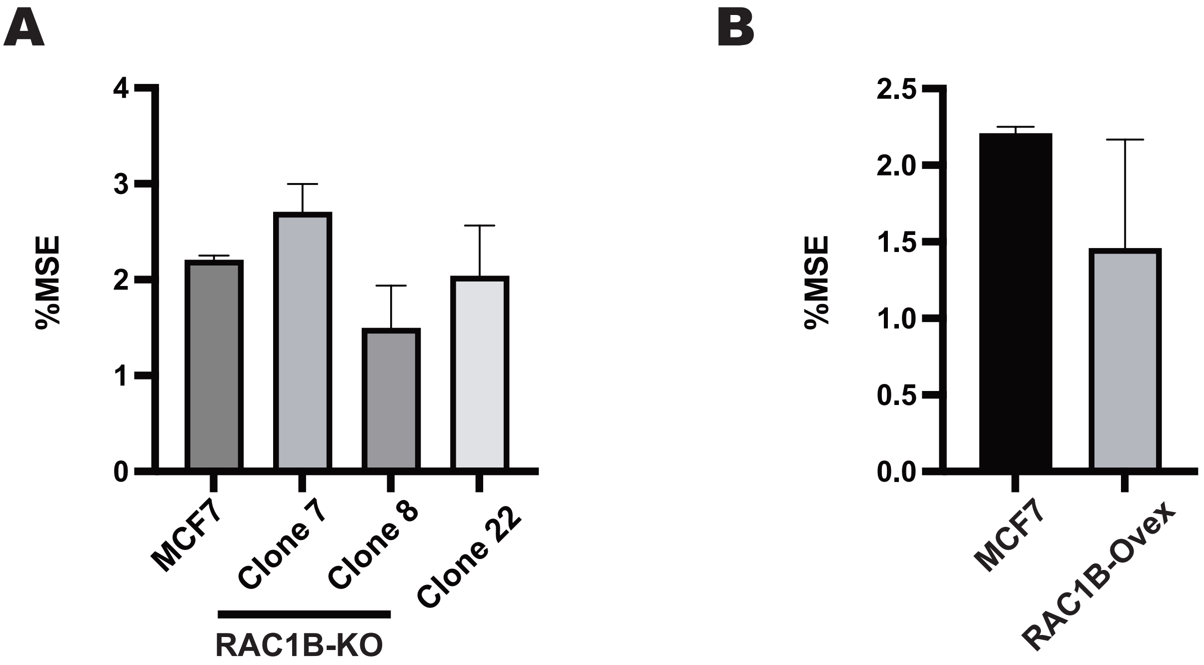

Supplementary Figure 3. Loss- or gain-of RAC1B function does not affect secondary mammosphere formation in MCF7 cells

A) Secondary mammosphere forming efficiency (%MSE) of parental MCF7, RAC1B-proficient Clone 22 (WT) and RAC1B-null single-cell clones were calculated by quantifying the number of secondary mammospheres formed per 100 cells plated for the primary mammosphere culture. Primary mammospheres were collected on day-5 of culture, dissociated into single cells and re-plated for secondary mammosphere formation for a further 7 day culture prior to counting. Values represent the mean ± SD of 3 independent experiments. No significant difference was observed between any clones compared to parental MCF7 group using paired t-test.

B) Secondary mammosphere forming efficiency (%MSE) of parental MCF7 and doxycycline-inducible RAC1B-overexpressing MCF7 clone was quantified using the same approach as in (**A**). Values represent the mean ± SD of 3 independent experiments. No significant difference was observed between samples using paired t-test.

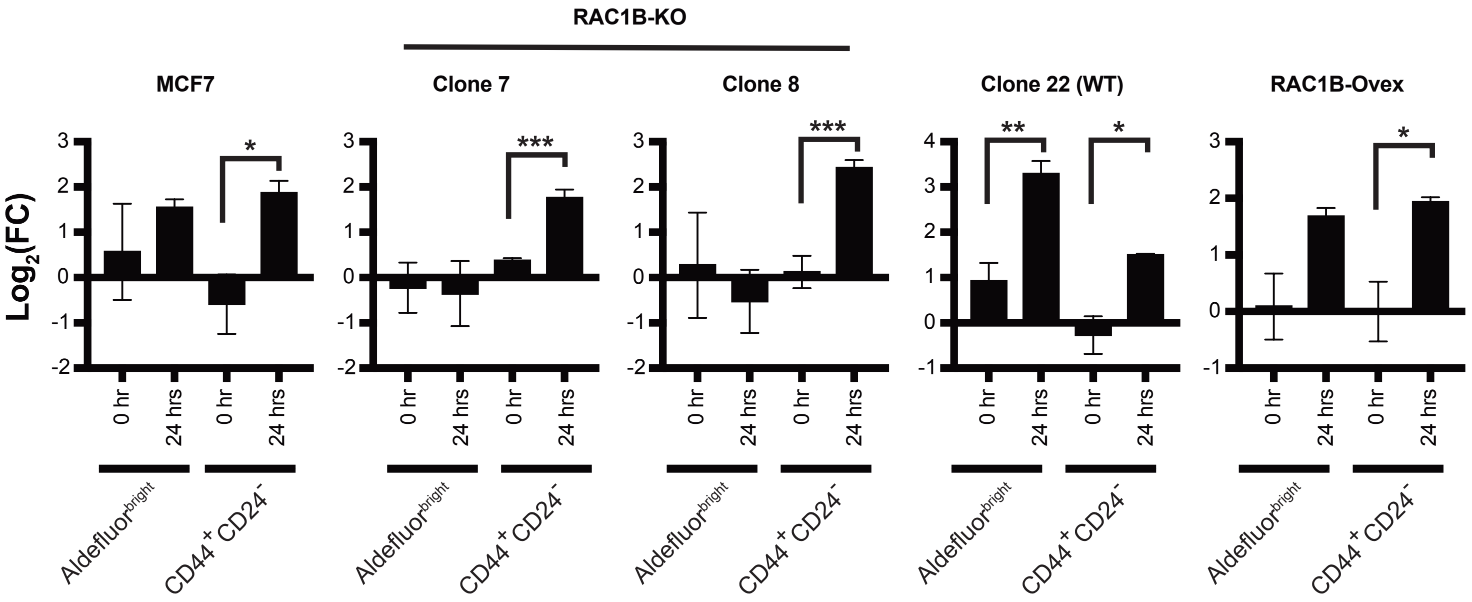

Supplementary Figure 4. RAC1B function alters the doxorubicin treatment-induced BCSC plasticity in MCF7 cells.

Fold change in the size of Aldefluorbright and CD44+;CD24- cell subpopulations in parental MCF7, RAC1B-proficient, RAC1B-null or RAC1B-overexpressing clones 0 hours or 24 hours after the end of 2.5 uM Doxorubicin treatment compared to their own untreated control groups. Fold change differences are shown in Log2 scale and represent the mean ± SEM of 3 independent experiments. (*:p<0.05, **:p<0.01, ***:p<0.005 two-tailed t-test).

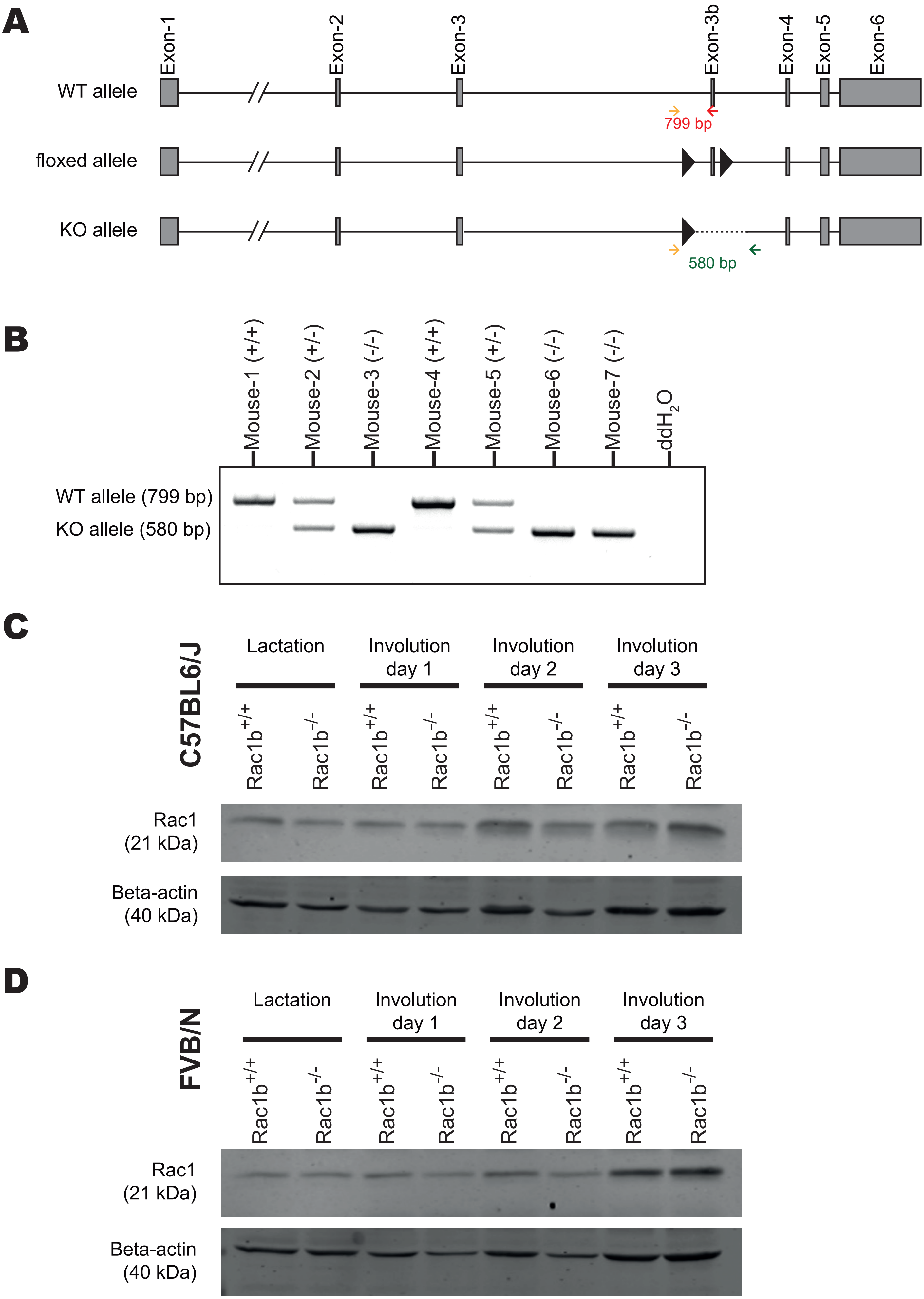

A
Exon-1
Exon-2
Exon-3
Exon-3b
Exon-4
Exon-5
Exon-6
WT allele
799 bp
floxed allele
KO allele
580 bp
B
Mouse-1 (+/+)
Mouse-2 (+/-)
Mouse-3 (-/-)
Mouse-4 (+/+)
Mouse-5 (+/-)
Mouse-6 (-/-)
Mouse-7 (-/-)
ddH2O
WT allele (799 bp)
KO allele (580 bp)
C
C57BL6/J
Lactation
Involution day 1
Involution day 2
Involution day 3
Rac1b+/+
Rac1b-/-
Rac1 (21 kDa)
Beta-actin (40 kDa)
D
FVB/N
Lactation
Involution day 1
Involution day 2
Involution day 3
Rac1b+/+
Rac1b-/-
Rac1 (21 kDa)
Beta-actin (40 kDa)

Supplementary Figure 5. Germline genomic deletion of exon3b results in the loss-of Rac1b function without altering Rac1 expression.

A) The exon-intron maps of the wildtype (WT), floxed, and knockout (KO) Rac1b alleles. Exons are shown as rectangles in gray. Black triangles show the location of the LoxP sites, whereas the dashed line in the KO allele is the region of genomic deletion obtained after germline deletion via using a universal-Cre deleter mouse line. The location of PCR primers used for genotyping are depicted as arrows with expected fragment sizes are shown for the WT and KO alleles.

B) A representative example of PCR results used for genotyping the Rac1b-KO mouse line.

C,D) Representative examples of immunoblot results for the expression of Rac1 in lactation and involution stage mammary glands of Rac1b+/+ and Rac1-/- mice in C57BL6/J (**C**) and FVB/N (**D**) backgrounds.

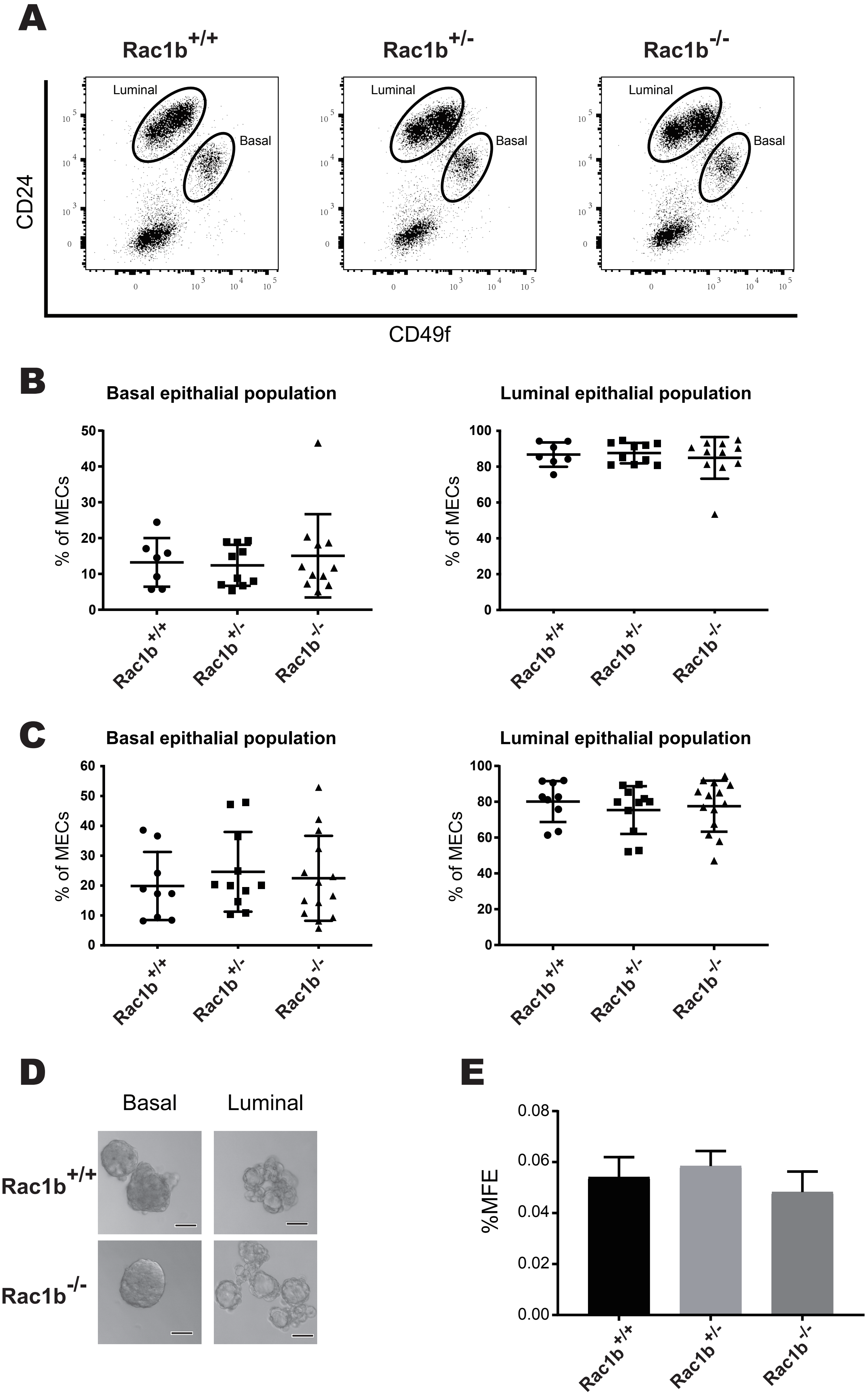
A
Rac1b+/+
Rac1b+/-
Rac1b-/-
Luminal
Basal
CD24
CD49f
B
Basal epithalial population
Luminal epithalial population
% of MECs
Rac1b +/+
Rac1b +/-
Rac1b -/-
C
Basal epithalial population
Luminal epithalial population
% of MECs
D
Basal
Luminal
Rac1b+/+
Rac1b-/-
E
%MFE
Rac1b +/+
Rac1b +/-
Rac1b -/-

Supplementary Figure 6. Rac1b function is dispensable for mammary epithelial lineage diversification and MaSC maintenance.

A) Representative dot plots for flow cytometry analysis of primary mammary cells isolated from nulliparous Rac1b$^{+/+}$, Rac1b$^{+/-}$, and Rac1b$^{-/-}$ mice. Basal and luminal mammary epithelial cells were identified based on their CD49f and CD24 expression levels within the alive, single, and lineage (CD45, TER119, CD31)-negative cell population.

B,C) Scatter plots showing the percentage distribution of mammary epithelial cells (MECs) into the basal and luminal mammary epithelial cell populations in the mammary glands of nulliparous Rac1b$^{+/+}$, Rac1b$^{+/-}$, and Rac1b$^{-/-}$ mice in C57BL6/J (**B**. n=7, 10, and 11, respectively) and FVB/J (**C**. n=11, 9, and 14, respectively) backgrounds. Lines represent the mean ± SD on scatter plots. There is no significant difference between genotypes as determined by unpaired t-test.

D) Representative images of mammospheres and acini structures formed by basal and luminal mammary epithelial cells, respectively, sorted from nulliparous Rac1b$^{+/+}$, and Rac1b$^{-/-}$ mice. Scale bars represent 50um.

E) Mammosphere forming efficiency (%MFE) of basal mammary epithelial cell populations isolated from nulliparous Rac1b$^{+/+}$ (n=4), Rac1b$^{+/-}$ (n=5), and Rac1b$^{-/-}$ (n=5) mice. Values represent the mean ± SD. There is no significant difference between genotypes as determined by unpaired t-test.

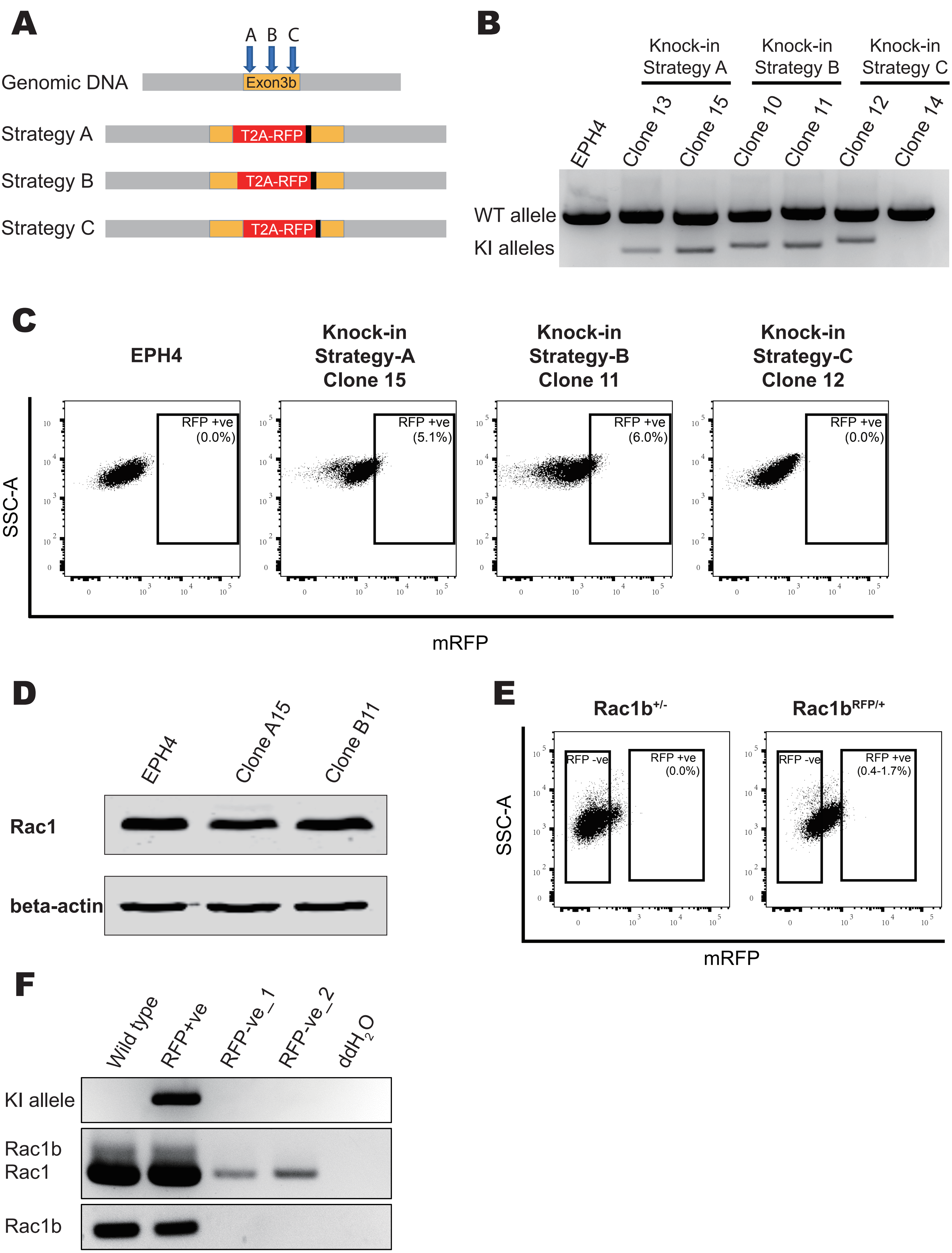
A
A B C
Genomic DNA
Exon3b
Strategy A
T2A-RFP
Strategy B
T2A-RFP
Strategy C
T2A-RFP
B
Knock-in Strategy A
Knock-in Strategy B
Knock-in Strategy C
EPH4
Clone 13
Clone 15
Clone 10
Clone 11
Clone 12
Clone 14
WT allele
KI alleles
C
EPH4
Knock-in Strategy-A Clone 15
Knock-in Strategy-B Clone 11
Knock-in Strategy-C Clone 12
RFP +ve (0.0%)
RFP +ve (5.1%)
RFP +ve (6.0%)
RFP +ve (0.0%)
SSC-A
mRFP
D
EPH4
Clone A15
Clone B11
Rac1
beta-actin
E
Rac1b+/-
Rac1bRFP/+
RFP -ve
RFP +ve (0.0%)
RFP -ve
RFP +ve (0.4-1.7%)
SSC-A
mRFP
F
Wild type
RFP+ve
RFP-ve_1
RFP-ve_2
ddH2O
KI allele
Rac1b
Rac1
Rac1b

Supplementary Figure 7. HDR-coupled CRISPR-mediated targeting to generate a novel transgenic mouse model as a surrogate reporter for Rac1b splicing.

A) Schematic representation of three alternative HDR template designs that would result in the in-frame insertion of the T2A-mRFP cassette close to the 5'-end (Strategy A), in the middle (Strategy B), or close to the 3'-end (Strategy C) of exon3b-encoding sequence. Approximate sites of insertions are shown with vertical blue arrows on exon3b-coding sequence, which is depicted in yellow and flanked by intronic sequences depicted in grey colour. T2A-RFP cassette is shown in red and the stop codons at the end of the transgene cassette are shown in black.

B) Genotyping PCR results of the genomic DNA obtained from single-cell EPH4 clones after HDR-coupled CRISPR targeting by using three different HDR templates as shown in (**A**). These results demonstrate that the knock-in (KI) was successful in all single-cell clones shown, except Clone 14 of Strategy C. Of note, the expected size of the KI allele band differs depending on the site of insertion of the T2A-mRFP cassette.

C) Flow cytometry analysis of selected clones demonstrate that RFP^+ cells constitute a small subpopulation of EPH4 cells only in clones with the knock-in of the T2A-mRFP cassette using HDR template of Strategy A or B as shown in (**A**). The RFP^+ gate was determined based on parental EPH4 cells.

D) Immunoblot analysis confirms that Rac1 protein levels were not affected by the knock-in of T2A-mRFP cassette in the successful single-cell KI clones.

E) Representative dot plots for flow cytometric analyses of primary mammary cells of nulliparous $Rac1b^{+/-}$ and $Rac1b^{RFP/+}$ mice are shown for the single alive cell population. The gate for RFP^+ cells was determined based on the signal in $Rac1b^{+/-}$ sample.

F) RT-PCR analysis of sorted cells as shown in (**E**). Wildtype corresponds to RFP^- cells sorted from the $Rac1b^{+/-}$ mammary glands; RFP^+ and RFP^- cells are sorted from $Rac1b^{RFP/+}$ mammary glands. RT-PCR for KI allele (upper panel) determines transcripts arising from the transgenic allele only; for Rac1/Rac1b (middle panel) can detect both Rac1 and Rac1b variants simultaneously; and for Rac1b (lower panel) is variant-specific to determine Rac1b variant only. ddH_2O is the negative control sample for the PCR.

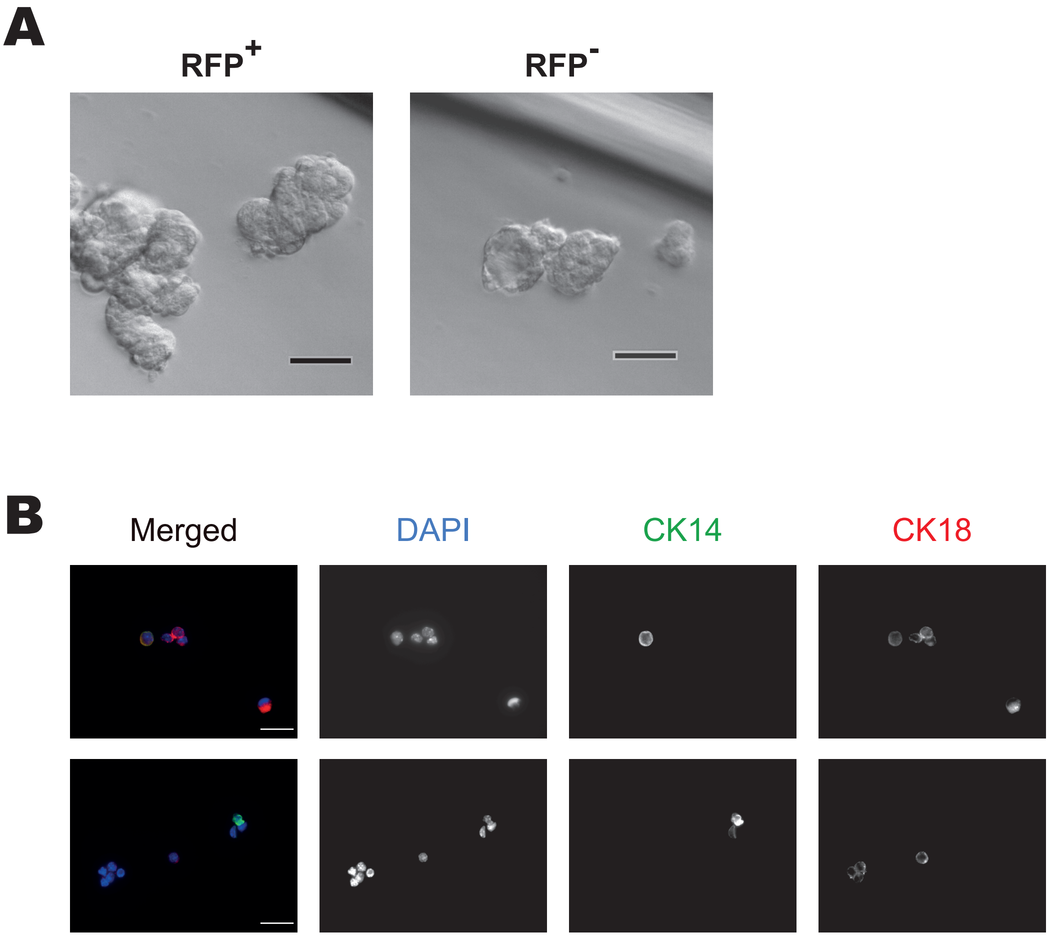

Supplementary Figure 8. Characterization of RFP^+ cell population isolated from $Rac1b^{RFP/+}$;MMTV-NIC mammary tumors.

A) Representative images of primary mammosphere cultures of Lin^-RFP^+ and Lin^-RFP^- cells sorted from $Rac1b^{RFP/+}$;MMTV-NIC tumors (n=6). Scale bars represent 50 um.

B) Representative images of CK14 and CK18 co-immunofluorescence staining of cyto-spinned Lin^-RFP^+ cells sorted from $Rac1b^{RFP/+}$;MMTV-NIC tumors (n=3). DAPI staining is used as a nuclear counterstain. Scale bars represent 20 um.

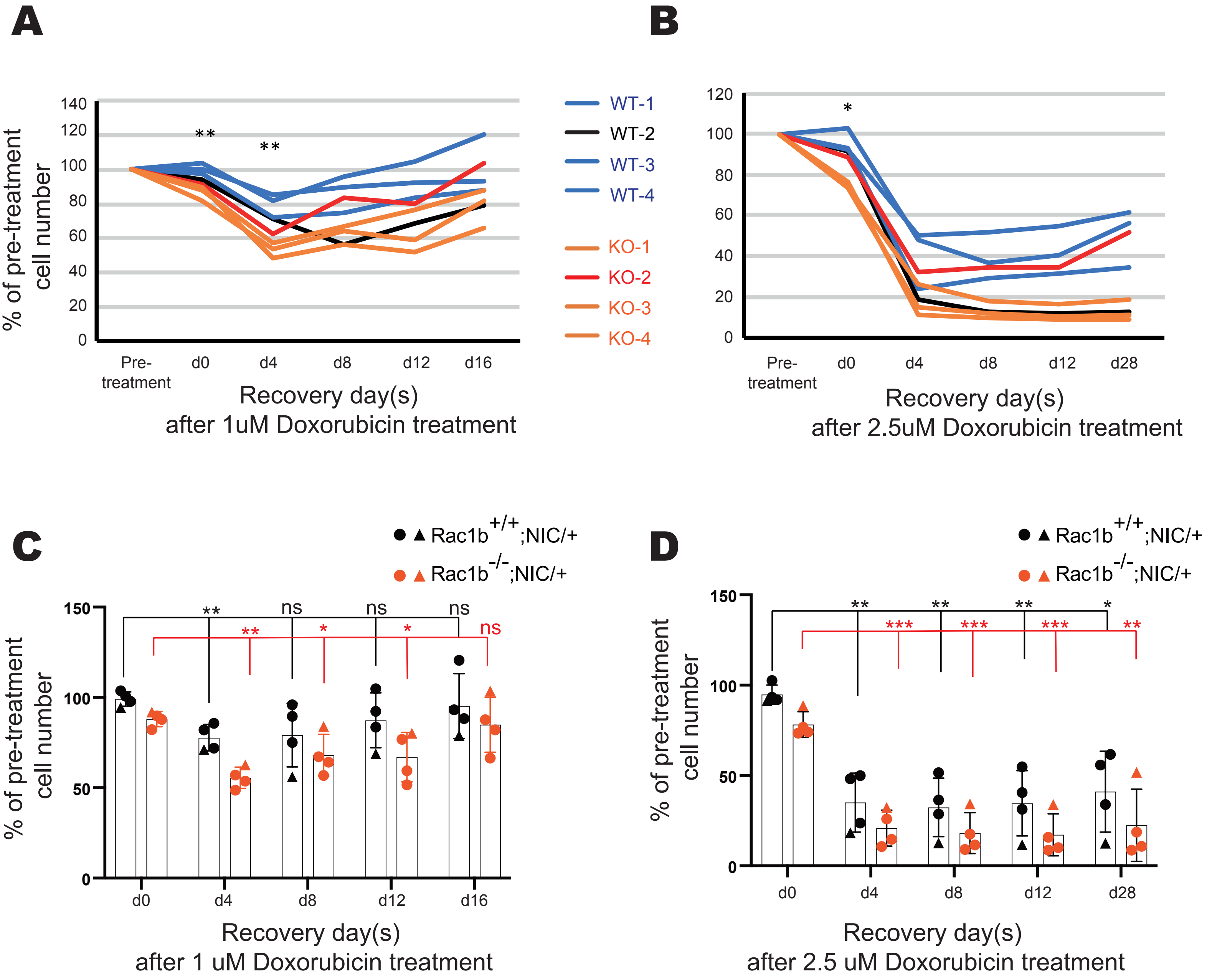

Supplementary Figure 9. Neu-driven primary tumor cell lines with loss-of Rac1b function shows increased likelihood of chemosensitivity to doxorubicin treatment.

A,**B**) Cell growth curve of Neu-driven primary tumor cell lines obtained from Rac1b$^{+/+}$;MMTV-NIC (n=4) and Rac1b$^{-/-}$;MMTV-NIC (n=4) mammary tumors in post-treatment recovery period after 1 uM (A) or 2.5 uM (B) doxorubicin treatment for 24 hours. Cell numbers are normalized to the pre-treatment cell number for each primary cell line and percentage changes are shown separately for each individual cell line with averages taken from 3 independent experiments. The outlier Rac1b-null chemoresistant cell line (KO-2) and the outlier Rac1b-proficient chemosensitive cell line (WT-2) is shown in red and black color, respectively. (*:$p<0.05$ and **:$p<0.01$, two-tailed unpaired t-test for comparison of KO versus WT).

C,**D**) Cell growth data shown in **A** and **B** are displayed as bar graphs representing mean ± SEM of 4 independent Rac1b-proficient or Rac1-null cell lines for time-dependent comparison of cell recovery. The data for each individual cell line is plotted on the bar graphs as circles or triangles, where triangles represent the data for the outlier cell lines WT-2 and KO-2. Post-treatment recovery was evaluated in comparison to the day-0 levels using two-way ANOVA and the comparative results are shown with black or red lines for Rac1b-proficient or Rac1b-null lines, respectively. (*:$p<0.05$, **:$p<0.01$, ***:$p<0.001$)

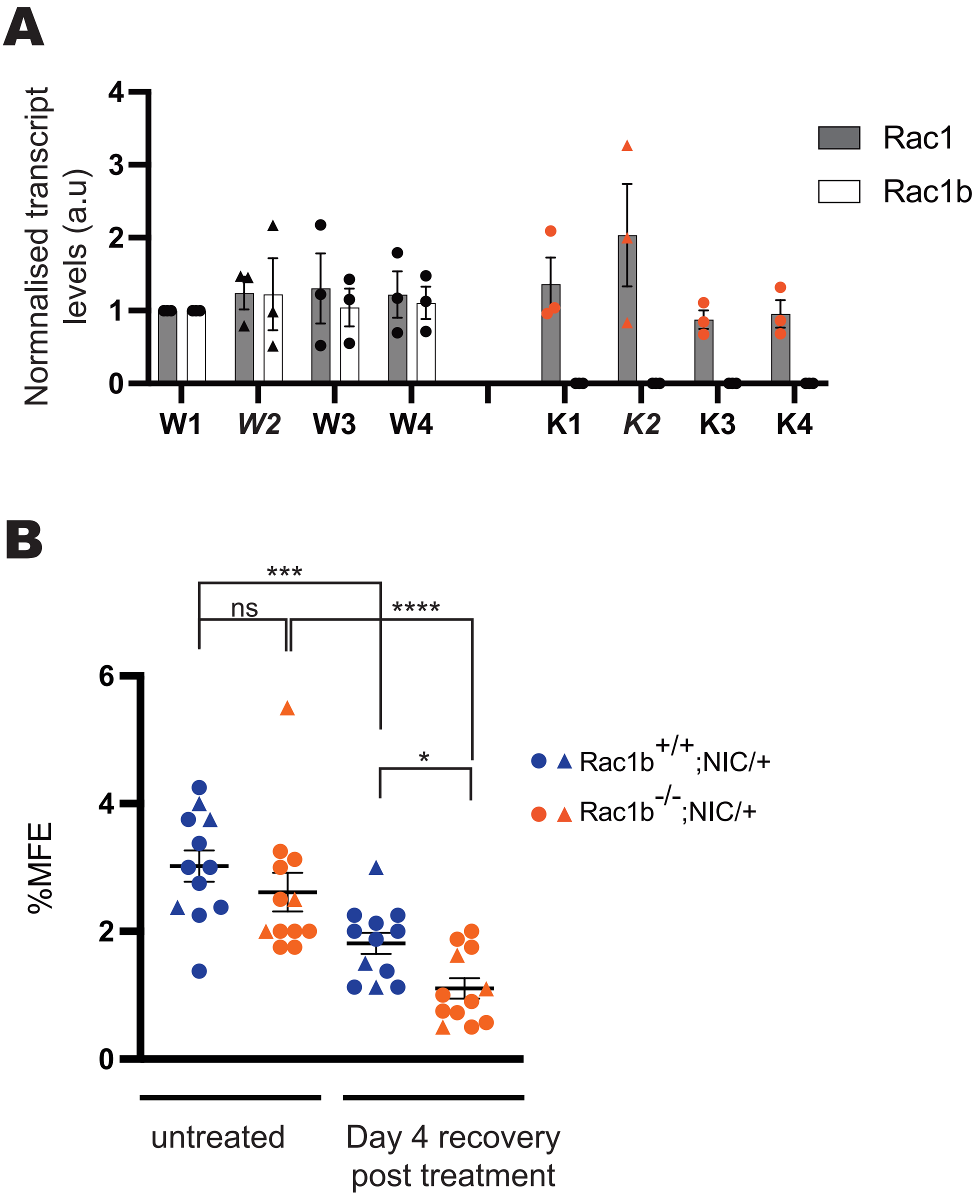

Supplementary Figure 10. Rac1/Rac1b expression levels and mammosphere forming efficiencies in Rac1b-proficient and Rac1b-null primary cell lines.

A) Quantitative RT-PCR results for Rac1 and Rac1b transcript levels in individual Rac1b-proficient and Rac1b-null primary cell lines. Values represent the mean ± SEM of 3 independent samples of each cell line and normalized to the transcript levels of one of the Rac1b-proficient line (i.e. W1) in each experiment. Individual data points show the results obtained in each sample tested, and the outlier chemosensitive Rac1b-proficient and chemoresistant Rac1b-null clones are shown as triangles, whereas others are shown as circles. No statistical difference was observed between any samples as tested by two-tailed paired t-test.

B) Scatter plot representation of the data shown in Figure 8C for the mammosphere forming efficiency (%MFE) of Rac1b$^{+/+}$;MMTV-NIC (n=4) and Rac1b$^{-/-}$;MMTV-NIC (n=4) primary tumor cell lines either in the absence of doxorubicin treatment or 4 days after 24 hours treatment with 2.5 uM doxorubicin. Results of the 3 independent experiments are shown separately as data points, but clustered together for n=4 cell lines of similar genotypes (12=4*3). However, the data for the outlier chemosensitive Rac1b-proficient (W2) and the chemoresistant Rac1b-null (K2) clones are shown with triangles for easier identification, whereas others are shown with circles. (ns: non-significant, **: p<0.01, ***: p<0.0005, ****: p<0.0001, two-way-ANOVA)

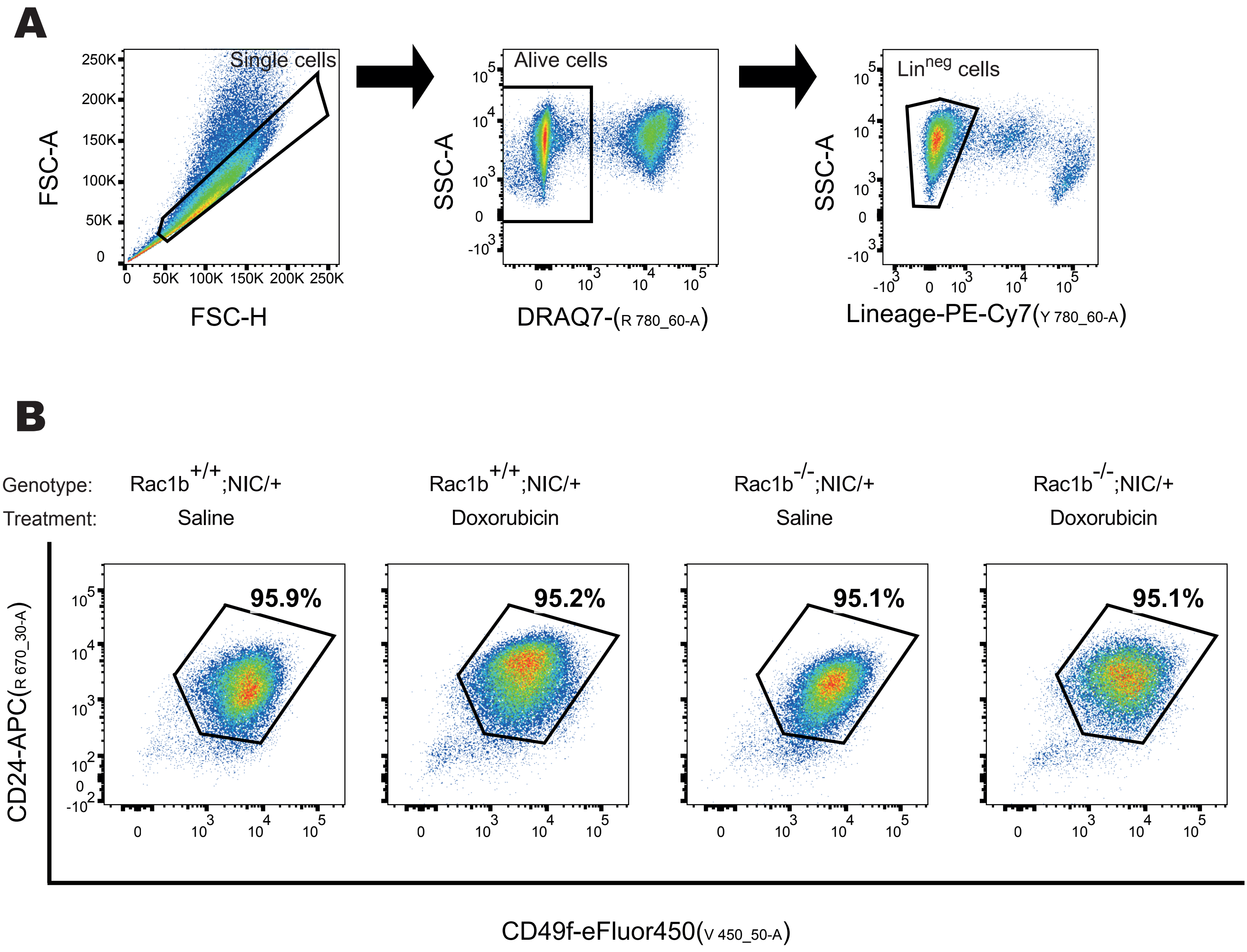

Supplementary Figure 11. Flow cytometry analysis and sorting of primary tumor epithelial cells.
A) Representative dot plots used for gating out cell clusters, dead cells and lineage-positive cells. Dissociated cells obtained from primary murine tumors were first gated to select single cells using forward scatter values – FSC-A versus FSC-H -, followed by the removal of DRAQ7-stained dead cells. Single alive cells were then gated to exclude Lineage-positive cells, which are CD45+ lymphocytes, TER119+ erythrocytes and CD31+ endothelial cells. SSC-A: Side scatter-Area
B) Representative dot plots used for identifying and sorting tumor epithelial cells based on their CD24 and CD49f expression. Tumor samples shown here are the ones isolated from saline- or doxorubicin-treated Rac1b$^{+/+}$;MMTV-NIC and Rac1b$^{-/-}$;MMTV-NIC mice.

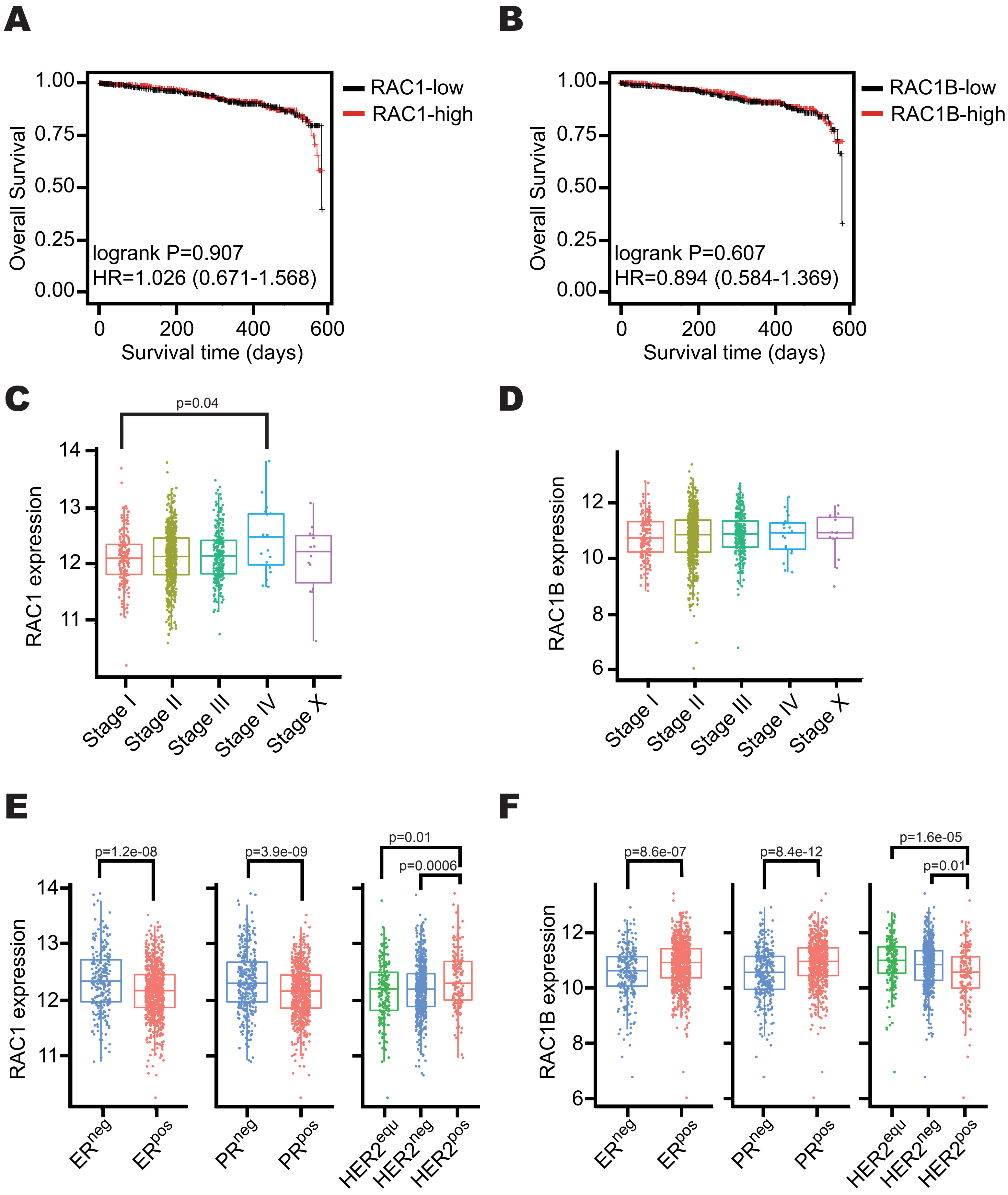
A
B
C
D
E
F
RAC1-low
RAC1-high
Overall Survival
logrank P=0.907
HR=1.026 (0.671-1.568)
Survival time (days)
RAC1B-low
RAC1B-high
logrank P=0.607
HR=0.894 (0.584-1.369)
p=0.04
RAC1 expression
RAC1B expression
Stage I
Stage II
Stage III
Stage IV
Stage X
p=1.2e-08
p=3.9e-09
p=0.01
p=0.0006
p=8.6e-07
p=8.4e-12
p=1.6e-05
p=0.01
ER^neg
ER^pos
PR^neg
PR^pos
HER2^equ
HER2^neg
HER2^pos

Supplementary Figure 12. TCGA dataset analyses for the identification of correlations between breast tumor characteristics and RAC1 or RAC1B expression levels.

A,B) Kaplan-Meier plots showing overall survival probability for breast cancer patients in TCGA dataset (n=993) stratified by RAC1 (**A**) or RAC1B (**B**) expression. Expression levels and survival data was retrieved from TSVdb. Hazard ratio (HR) and respective confidence intervals are shown. P values are determined by Log Rank test.

C,D) Scatterplot representation of RAC1(**C**) or RAC1B (**D**) transcript levels in breast tumors within TCGA dataset across pathological stages: Stage I (n=181), Stage II (n=612), Stage III (n=247), Stage IV (n=19), and Stage X (n=14).

E,F) Scatterplot representation of RAC1(**E**) or RAC1B (**F**) transcript levels in breast tumors within TCGA dataset across clinical staining subgroups: ER^{neg} (n=237), ER^{pos} (n=794), PR^{neg} (n=340), PR^{pos} (n=688), $HER2^{equivocal(equ)}$ (n=177), $HER2^{neg}$ (n=555), and $HER2^{pos}$ (n=161).

In **C-F**, expression levels are in log2 transformed normalised RSEM. Two-tailed student t-test and ANOVA with post-hoc Tukey HSD for multiple comparison were used to calculate statistical significances, and p values are shown on graphs for comparisons that show statistical significance ($p<0.05$).

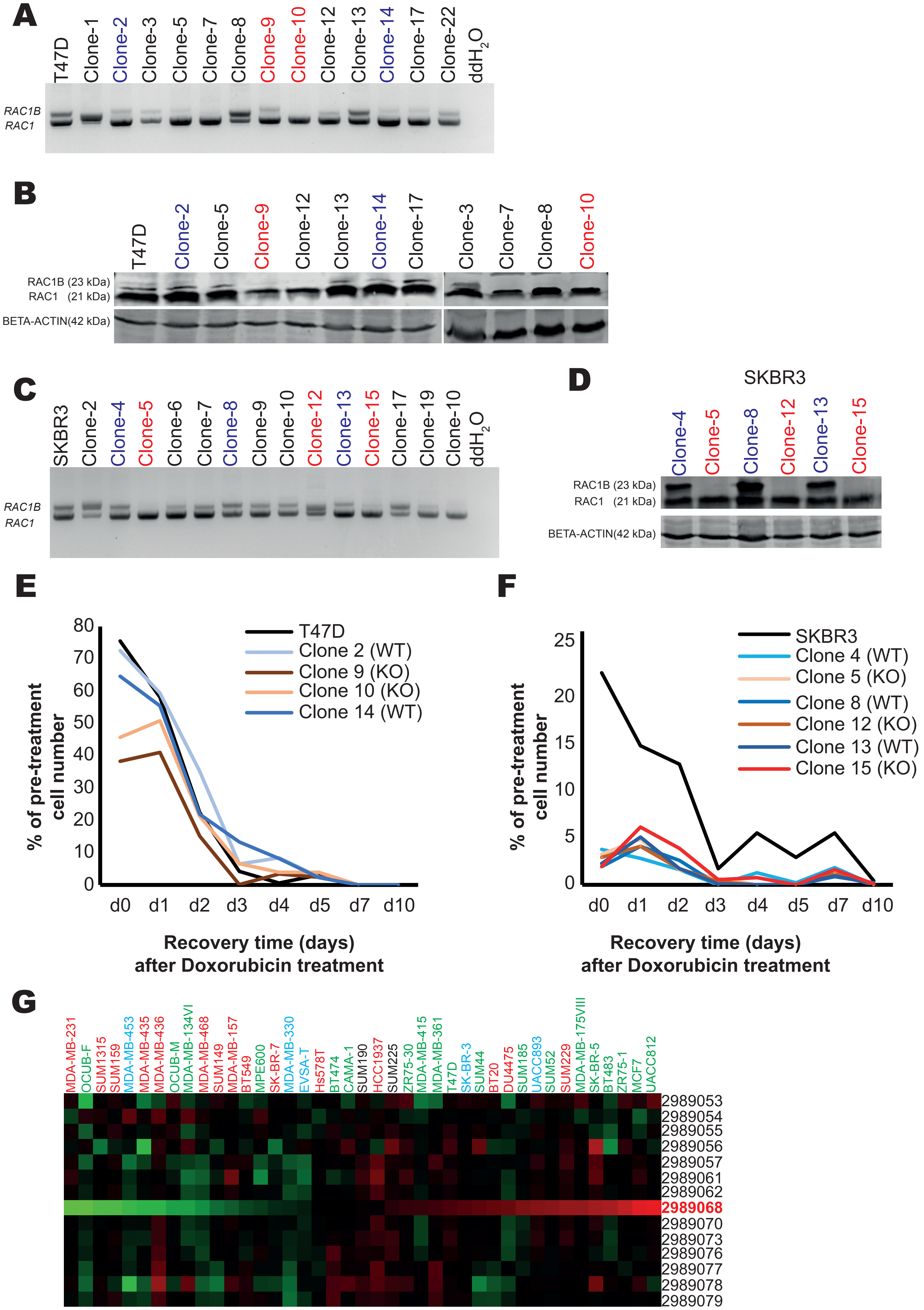
A
T47D
Clone-1
Clone-2
Clone-3
Clone-5
Clone-7
Clone-8
Clone-9
Clone-10
Clone-12
Clone-13
Clone-14
Clone-17
Clone-22
ddH2O
RAC1B
RAC1
B
T47D
Clone-2
Clone-5
Clone-9
Clone-12
Clone-13
Clone-14
Clone-17
Clone-3
Clone-7
Clone-8
Clone-10
RAC1B (23 kDa)
RAC1 (21 kDa)
BETA-ACTIN(42 kDa)
C
SKBR3
Clone-2
Clone-4
Clone-5
Clone-6
Clone-7
Clone-8
Clone-9
Clone-10
Clone-12
Clone-13
Clone-15
Clone-17
Clone-19
Clone-10
ddH2O
RAC1B
RAC1
D
SKBR3
Clone-4
Clone-5
Clone-8
Clone-12
Clone-13
Clone-15
RAC1B (23 kDa)
RAC1 (21 kDa)
BETA-ACTIN(42 kDa)
E
% of pre-treatment cell number
0
10
20
30
40
50
60
70
80
d0
d1
d2
d3
d4
d5
d7
d10
Recovery time (days) after Doxorubicin treatment
T47D
Clone 2 (WT)
Clone 9 (KO)
Clone 10 (KO)
Clone 14 (WT)
F
% of pre-treatment cell number
0
5
10
15
20
25
d0
d1
d2
d3
d4
d5
d7
d10
Recovery time (days) after Doxorubicin treatment
SKBR3
Clone 4 (WT)
Clone 5 (KO)
Clone 8 (WT)
Clone 12 (KO)
Clone 13 (WT)
Clone 15 (KO)
G
MDA-MB-231
OCUB-F
SUM1315
SUM159
MDA-MB-453
MDA-MB-435
MDA-MB-436
OCUB-M
MDA-MB-134VI
MDA-MB-468
SUM149
MDA-MB-157
BT549
MPE600
SK-BR-7
MDA-MB-330
EVSA-T
Hs578T
BT474
CAMA-1
SUM190
HCC1937
SUM225
ZR75-30
MDA-MB-415
MDA-MB-361
T47D
SK-BR-3
SUM44
BT20
DU4475
SUM185
UACC893
SUM52
SUM229
MDA-MB-175VIII
SK-BR-5
BT483
ZR75-1
MCF7
UACC812
2989053
2989054
2989055
2989056
2989057
2989061
2989062
2989068
2989070
2989073
2989076
2989077
2989078
2989079

Supplementary Figure 13. Loss-of RAC1B function does not alter the chemosensitivity to doxorubicin treatment in T47D and SKBR3 cells

A-D) RT-PCR (**A,C**) and immunoblot (**B,D**) analyses of T47D (**A,B**) and SKBR3 (**C,D**) single-cell CRISPR clones. Beta-Actin was used as a loading control in immunoblots. RAC1B-proficient (WT) and RAC1B-null (KO) clones selected for phenotype analyses are shown in blue and red colors, respectively.

E,F) Cell growth curve of parental, RAC1B-proficient (WT) and RAC1B-null (KO) single-cell clones of T47D (**E**) and SKBR3 (**F**) cells in post-treatment recovery period after 2.5 uM doxorubicin treatment for 48 hours. Cell numbers are presented as percentage of pre-treatment cell numbers for each individual clone. Each data point represents the mean of three independent experiments. No significant differences were observed when RAC1B-null clones were compared to RAC1B-proficient clones with two-tailed unpaired t-test.

G) A previously published exon array dataset for 40 different breast cancer cell lines [48] was analyzed to stratify them for their normalized RAC1B transcript levels. The probe 2989068 corresponds to the exon3b-encoded transcript sequence. Cell lines' names were written on the heatmap in different colors to indicate their intrinsic molecular subtypes: Red, triple-negative breast cancer; Blue, HER2+ breast cancer; green, Luminal breast cancer.
